# A Diffusion-like Process Accommodates New Crypts During Clonal Expansion in Human Colonic Epithelium

**DOI:** 10.1053/j.gastro.2021.04.035

**Published:** 2021-08

**Authors:** Cora Olpe, Doran Khamis, Maria Chukanova, Nefeli Skoufou-Papoutsaki, Richard Kemp, Kate Marks, Cerys Tatton, Cecilia Lindskog, Anna Nicholson, Roxanne Brunton-Sim, Shalini Malhotra, Rogier ten Hoopen, Rachael Stanley, Douglas J. Winton, Edward Morrissey

**Affiliations:** 1 Cancer Research-UK Cambridge Institute, Li Ka Shing Centre, Robinson Way, Cambridge, United Kingdom; 2 Wellcome Trust-Medical Research Council, Cambridge Stem Cell Institute, Cambridge, UK; 3 MRC Weatherall Institute of Molecular Medicine, University of Oxford, John Radcliffe Hospital, Headington, Oxford, United Kingdom; 4 Pathology and Data Analytics, St James’s University Hospital, Leeds, United Kingdom; 5 Department of Immunology, Genetics and Pathology, Science for Life Laboratory, Rudbeck Laboratory, Uppsala University, Uppsala, Sweden; 6 Norwich Research Park BioRepository, Norwich, United Kingdom; 7 Department of Oncology, University of Cambridge, Cambridge, United Kingdom

**Keywords:** Colorectal Cancer Initiation, Intestinal Crypt, Crypt Fission, Crypt Fusion, bp, base pair, CI, confidence interval, CRC, colorectal cancer, FFPE, formalin-fixed paraffin-embedded, FUFI, crypt fusion or fission form, *χ*, contribution of mutant FUFIs to fusions, MAF, mutant allele frequency, M/M, mutant/mutant, M/W, mutant/wild-type, PCR, polymerase chain reaction, PPC, partially populated crypt, WPC, wholly populated crypt

## Abstract

**Background & Aims:**

Colorectal cancer (CRC) is thought to arise when the cumulative mutational burden within colonic crypts exceeds a certain threshold that leads to clonal expansion and ultimately neoplastic transformation. Therefore, quantification of the fixation and subsequent expansion of somatic mutations in normal epithelium is key to understanding colorectal cancer initiation. The aim of the present study was to determine how advantaged expansions can be accommodated in the human colon.

**Methods:**

Immunohistochemistry was used to visualize loss of the cancer driver KDM6A in formalin-fixed paraffin-embedded (FFPE) normal human colonic epithelium. Combining microscopy with neural network-based image analysis, we determined the frequencies of KDM6A-mutant crypts and fission/fusion intermediates as well as the spatial distribution of clones. Mathematical modeling then defined the dynamics of their fixation and expansion.

**Results:**

Interpretation of the age-related behavior of KDM6A-negative clones revealed significant competitive advantage in intracrypt dynamics as well as a 5-fold increase in crypt fission rate. This was not accompanied by an increase in crypt fusion. Mathematical modeling of crypt spacing identifies evidence for a crypt diffusion process. We define the threshold fission rate at which diffusion fails to accommodate new crypts, which can be exceeded by KRAS activating mutations.

**Conclusions:**

Advantaged gene mutations in KDM6A expand dramatically by crypt fission but not fusion. The crypt diffusion process enables accommodation of the additional crypts up to a threshold value, beyond which polyp growth may occur. The fission rate associated with *KRAS* mutations offers a potential explanation for *KRAS*-initiated polyps.

## Graphical abstract

**Figure:**
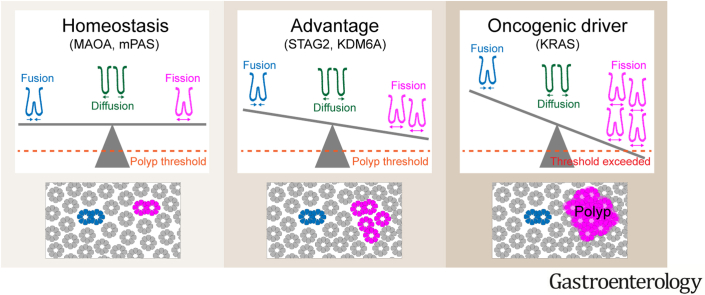

**What You Need to Know**

**Background and Context**

As colorectal cancer is thought to arise from outgrowth of crypts harboring excess genomic alterations, quantification of mutation accumulation and spread within this tissue is key to understanding disease initiation.

**New Findings**

Exploiting loss of the cancer driver KDM6A, we demonstrate that newly generated crypts resulting from increased crypt fission are accommodated by mass movement of surrounding crypts in a diffusion-type process.

**Limitations**

Only 2 cancer driver genes, KDM6A and KRAS, are modeled here, which does not preclude the existence of alternative expansion mechanisms.

**Impact**

The threshold fission rate beyond which diffusion cannot accommodate newly generated crypts is calculable defining when identifiable pathologies may form.

It is widely recognized that many renewing epithelia acquire a substantial burden of cancer driver mutations while remaining apparently normal.^1^, ^2^, ^3^ In the human colon, development of neoplastic disease is thought to be driven by elevated rates of gland replication or fission. Most notably, loss of the tumor suppressor gene APC generates adenomas in this way.^4^, ^5^, ^6^, ^7^ Yet throughout life, normal crypts also undergo crypt replication at a low rate, which can be elevated by advantaged mutations.^8^, ^9^, ^10^, ^11^, ^12^, ^13^, ^14^, ^15^, ^16^, ^17^

There does not appear to be an increase in the net density of crypts, or of colonic epithelial area, with age.^18^ This raises the question: how are local clonal expansions arising from elevated fission rates accommodated? One explanation might lie in crypt fusion. This process has recently been described and could counteract the consequences of fission.^19^^,^^20^ However, it remains unclear if fusion is an independent stochastic process or if it is locally coregulated with fission. The latter possibility may be particularly relevant to pro-oncogenic mutations as fusions at the edge of mutant patches could enable effective local invasion of wild-type crypts with a high probability of subsequent displacement of wild-type cells.

*KDM6A* (UTX) is an X-linked gene encoding a histone demethylase that specifically targets di- and tri-methyl groups on lysine 27 of histone H3. Inactivating mutations and deletions of *KDM6A* have been identified in a variety of human cancers, including colon, bladder, prostate, and esophageal cancer.^21^, ^22^, ^23^, ^24^ *KDM6A* featured among the 127 significantly mutated genes in The Cancer Genome Atlas study that analyzed 3281 tumors derived from 12 cancer types.^25^ *KDM6A* mutations are infrequent in CRCs (<4% of all tumors, COSMIC database).

Here, in seeking additional cancer driver events that can be visualized as somatic clones, we identify loss of *KDM6A* as possessing advantage in both intracrypt fixation and subsequent expansion. The large multicrypt clones resulting from elevated rates of crypt fission are investigated to study the impact of expansion on crypt packing and the role of crypt fusion in relieving overcrowding. The increased fission rate within *KDM6A*^−^ clones is not accompanied by an increase in crypt fusion, suggesting the 2 processes are driven by independent mechanisms and that fusion does not act to relieve local overcrowding. Instead, it is demonstrated that new crypts generated by fission can be accommodated by localized crypt diffusion up to a threshold beyond which hyperplastic and neoplastic lesions may form.

## Materials and Methods

### Human Tissue

Normal colon tissue samples were obtained from Addenbrooke’s Hospital Cambridge and Norfolk and Norwich University Hospital under full local research ethical committee approval (Documents 15/WA/0131 and 17/EE/0265, and 06/Q0108/307 and 08/H0304/85, respectively) according to UK Home Office regulations. The study included 273 individuals aged 13 to 93 years. Colectomy specimens were fixed in 10% neutral buffered formalin. From areas without macroscopically visible disease, mucosal sheets were removed and embedded en face in paraffin blocks. Sections were cut at 5-μm thickness onto charged glass slides.

### Histochemistry

#### mPAS staining

This was performed as previously published.^26^

#### Immunohistochemistry

Antibodies are listed in Supplementary Table 1. For standard sections, immunohistochemistry was performed as previously published.^26^ For laser capture microdissection, tissue was cut at 10-μm thickness onto UV-irradiated PEN membrane slides (Zeiss, Oberkochen, Germany). Heat-induced epitope retrieval was performed in citrate buffer in a water bath at 76 °C for 16 hours. Counterstaining with Mayer’s Hematoxylin was performed manually for 15 seconds followed by blueing in tap water for 1 minute.

### Experimental Pathology

#### Data acquisition

Clones were scored by brightfield microscopy, followed by scanning of sections using a Leica (Wetzlar, Germany) Aperio AT2 scanner. The DeCryptICS image analysis tool developed by Edward Morrissey and Doran Khamis (https://github.com/MorrisseyLab/DeCryptICS) was used to count total number of crypts as well as Fusion or Fission (FUFI) forms per section. This was followed by manual quality control using QuPath^27^ including classification of FUFIs into mutant/mutant (M/M), mutant/wild-type (M/W), or entirely wild-type.

#### Quality control

Within the dataset, 2 individuals aged 37 years with extreme average patch sizes were identified as outliers with respect to that measure and not included in subsequent analyses of patch sizes, fusion rates, and newly generated crypts.

### DNA Extraction From FFPE Tissue

#### Laser capture microdissection

Crypts were harvested into lids of 0.2-mm radius polymerase chain reaction (PCR) tubes using a Leica LMD7000 Laser Microdissection System; 10 μL of Proteinase K solution from the Arcturus PicoPure DNA Extraction Kit (ThermoFisher, Waltham, MA) was added followed by lysis at 65 °C for 3 hours and inactivation at 95 °C for 10 minutes.

#### Extraction from sections for KRAS sequencing

The QIAmp DNA FFPE Tissue Kit (Qiagen, Hilden, Germany) was used according to the supplier’s protocol.

### Library Preparation and Sequencing

Primers, PCR reaction components, cycling conditions, and processing for amplification are described in Supplementary Table 2, Supplementary Table 3, Supplementary Table 4, Supplementary Table 5, Supplementary Table 6. Samples were barcoded using the Fast Start High Fidelity PCR System (Roche, Basel, Switzerland) according to the supplier’s protocol. After pooling and purification by Clean & Concentrator Kit (Zymo Research, Irvine, CA) and size selection by PippinBlue (Sage Science, Beverly, MA), samples were sequenced using 150-base pair (bp) paired-end sequencing with 10% PhiX in-house on the Illumina (San Diego, CA) MiSeq platform.

### Sequencing Data Analysis

Scripts are available under the following: https://github.com/kemp05/.

#### KDM6A

Amplicons were extracted by starting, finishing, and containing the expected sequence in the middle. Then, at every nucleotide position excluding the primer, the number of reads corresponding to the reference genome as well as those containing a base change were recorded. This enabled calculation of the noise at every position (Supplementary Table 7). Candidate mutations were identified when the mutant allele frequency (MAF) was either >4 times the mean of the noise at that position or >3.29 times the standard deviation at that position (*P ≤* .001). True mutations were called if present in all samples originating from the same patch in serial sections but absent in all wild-type samples from the same sections.

#### KRAS

Corresponding forward and reverse reads were combined using PANDAseq 2.11 with default options.^28^ Amplicons were extracted by beginning and ending with the expected primer sequence and correct overall length (± 3 bp tolerance).

For codon 12 and 13 mutation calling, reads containing the sequences corresponding to wild-type as well as all possible mutations (Supplementary Table 8) were extracted. This yielded an MAF for every possible mutation in all 4 amplicons for every sample and revealed the noise. KRAS mutations were called if (1) >1000 reads were obtained for both KRAS amplicons and at least 1 mimic amplicon, and (2) the MAF in both KRAS amplicons was > 0.1% (corresponding to at least 10 mutant reads) but found at background levels in the mimic amplicons. These criteria correspond to ≥1.96 standard deviations or a *P* value of < .025 for the noisiest nucleotide position (G12D). The actual MAF for a particular mutation was calculated by subtracting the mean allele frequency (noise).

### Mathematical Modeling

#### Stem cell dynamics and crypt fission

The stem cell dynamics and fission rate associated with loss of KDM6A were mathematically modeled as previously described.^26^

#### Crypt fusion and diffusion

Crypt fusion was modeled as a process parallel to fission, with the same duration. Therefore, the following applied:

$$\frac{number\;of\;fission\;events}{number\;of\;fusion\;events}\;∼\;\frac{fission\;rate}{fusion\;rate}$$

All M/W FUFIs were considered fusions, but M/M FUFIs could be fissions or fusions. Therefore, the number of fission events and fusion events required for the preceding equation were not directly measurable. However, they were calculable by sampling FUFIs from the edge of mutant patches, which enabled calculation of the proportion of M/M FUFIs that are fusions (termed chi: *χ*) by accounting for the distribution of the mutational state of neighboring crypts (see supplemental materials for mathematical details).

To infer a diffusion coefficient, growth of a patch through initial mutation and subsequent fissions was modeled as a stochastically firing point source of mass at the clone centroid. Potential trajectories from mutation hit to patch of size 10 (i.e, stochastic event times) were simulated, and an ensemble diffusion coefficient was inferred by randomly drawing paths from the set of simulated potential trajectories. The inferred diffusion coefficient was then used in a theoretical study of patch expansion (see supplemental materials for mathematical details).

#### *KRAS* expansion

The data obtained here were combined with our previously published dataset and analyzed as described there.^26^

## Results

### KDM6A-negative Clones Are Advantaged in Stem Cell Competition

**Figure 1:**
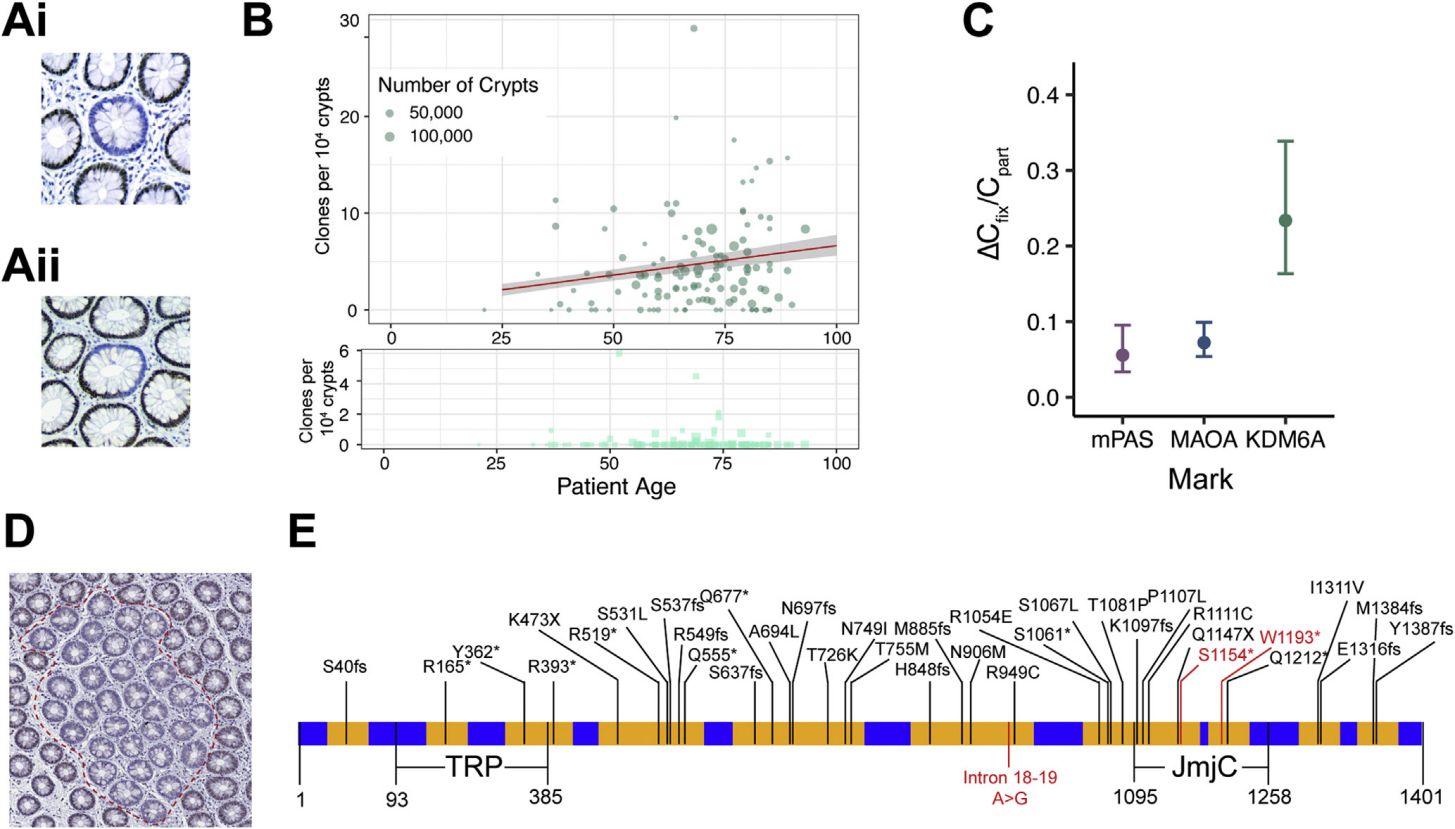
Detection of clonal loss of KDM6A in normal human colon. (*A*) Representative images of KDM6A^−^ WPC (*i*) and PPC (*ii*). (*B*) *Top*: Frequency plot showing age-related behaviors of KDM6A^−^ WPC (*circles*, *darker*) and PPC (*squares*, *lighter*). *Red line*: regression analysis showing accumulation of WPC (slope of accumulation of fixed clones [ΔC_*fix*_]: 6.04 × 10^−6^ per year) and 95% CI in *gray*. *Bottom*: PPC only on expanded y-axis. (*C*) Plot showing increased ratio of ΔC_fix_/frequency of partial clones (C_part_) for KDM6A (0.23, 95% CI 0.16–0.34) as compared with the neutral marks mPAS and MAOA (replotted from Nicholson et al^26^). Error bars = 95% CI. (*D*) Representative image of large KDM6A^−^ multicrypt patch highlighted by *dashed line*. (*E*) KDM6A cDNA structure annotated with sequenced areas (*yellow*), mutations found in COSMIC and mutations identified in KDM6A^−^ patches (*red*).

**Supplementary Figure 1:**
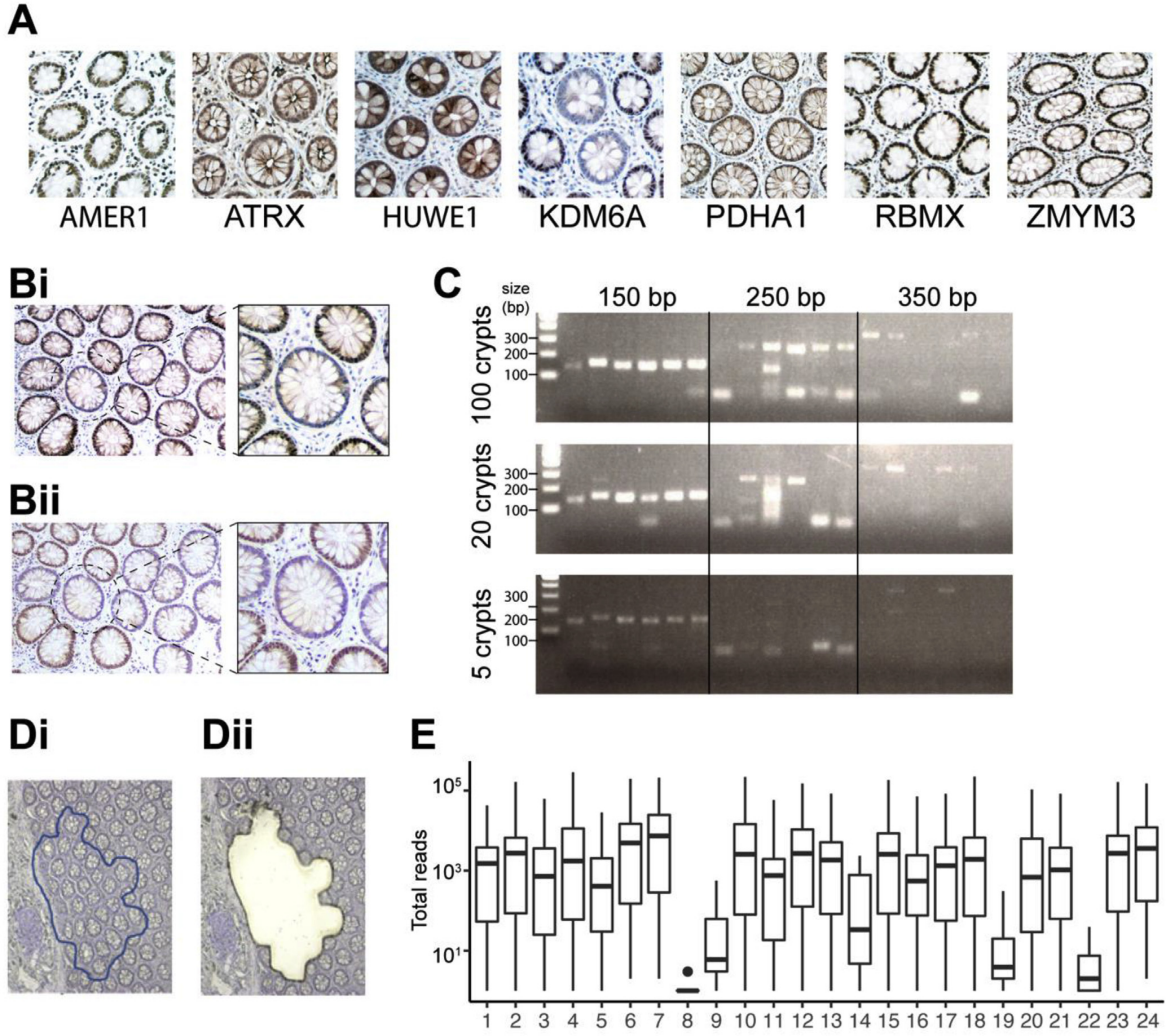
Detection and laser capture of KDM6A^−^ clones. (*A*) Representative images of immunohistochemistry (IHC) for cancer-associated X-linked genes on human colonic sections. Of the 7 assessed genes, a staining pattern indicative of loss-of-function mutation was only found for KDM6A (*middle*). (*B*) Serial human colonic sections stained with 2 independent antibodies against KDM6A: (*i*) human protein atlas, (*ii*) Cell Signaling Technology. KDM6A^−^ crypts are circled and enlarged. (*C*) For assessment of optimal amplicon size, DNA extracted from FFPE sections was diluted to equivalents of 5, 20, and 100 crypts and used for PCR reactions (primers in Supplementary Table 2). Image of gel electrophoresis of PCR products on FFPE DNA diluted to equivalents of varying amounts of crypts. This revealed that an amplicon size of 150 bp ensures reliable amplification. (*D*) Image of IHC for KDM6A on laser capture slide (*i*) before and (*ii*) after laser capture microdissection. (*E*) Box plot showing reads obtained for each of the 24 amplicons used for sequencing of KDM6A^−^ and control patches. Amplicon libraries from 4 patches failed quality control. Of the remaining 7 patches, mutations were identified in 4 at mutant allele frequencies consistent with patient sex and estimated stromal fraction within the captured material (Supplementary Table 4). These results both confirm antibody specificity for KDM6A and the clonality of multicrypt patches that share only a single mutation.

We have previously used visualization of loss of X-linked genes as clonal marks to quantify human colonic stem cell dynamics.^26^ In attempting to expand this methodology to X-linked genes with cancer association, clonal loss of KDM6A was identified by immunohistochemistry with 2 independent antibodies on normal human colonic epithelia (Supplementary Figure 1*A* and *B*). Intracrypt dynamics that describe the accumulation of clones wholly populating entire crypts (WPC) from partly populated (PPC) transition forms were determined for KDM6A^-^ clones using colonic FFPE sections from 120 patients aged 21 to 93 as previously described^26^ (Figure 1*A* and *B*). This revealed that loss of KDM6A confers a competitive advantage to affected stem cells shown as a decrease in the fraction of PPC supporting the accumulation of WPC (Figure 1*C*).

### KDM6A-negative Clones Expand by 5-Fold Increased Crypt Fission

Expansion of individual KDM6A^−^ clones was recognizable as large patches that frequently exceeded 10 crypts (Figure 1*D*). To confirm the clonal origin of such patches, we used laser capture microdissection followed by targeted sequencing that covered 3.6 kb of exonic and flanking intronic sequence of *KDM6A* (24 amplicons), including sites frequently mutated in human cancers. No patch was found to carry more than 1 *KDM6A* mutation and mutant allele frequencies were in line with predictions from patient sex and stromal content, supporting clonality (Figure 1*E*, Supplementary Figure 1*C*–*E*).

**Figure 2:**
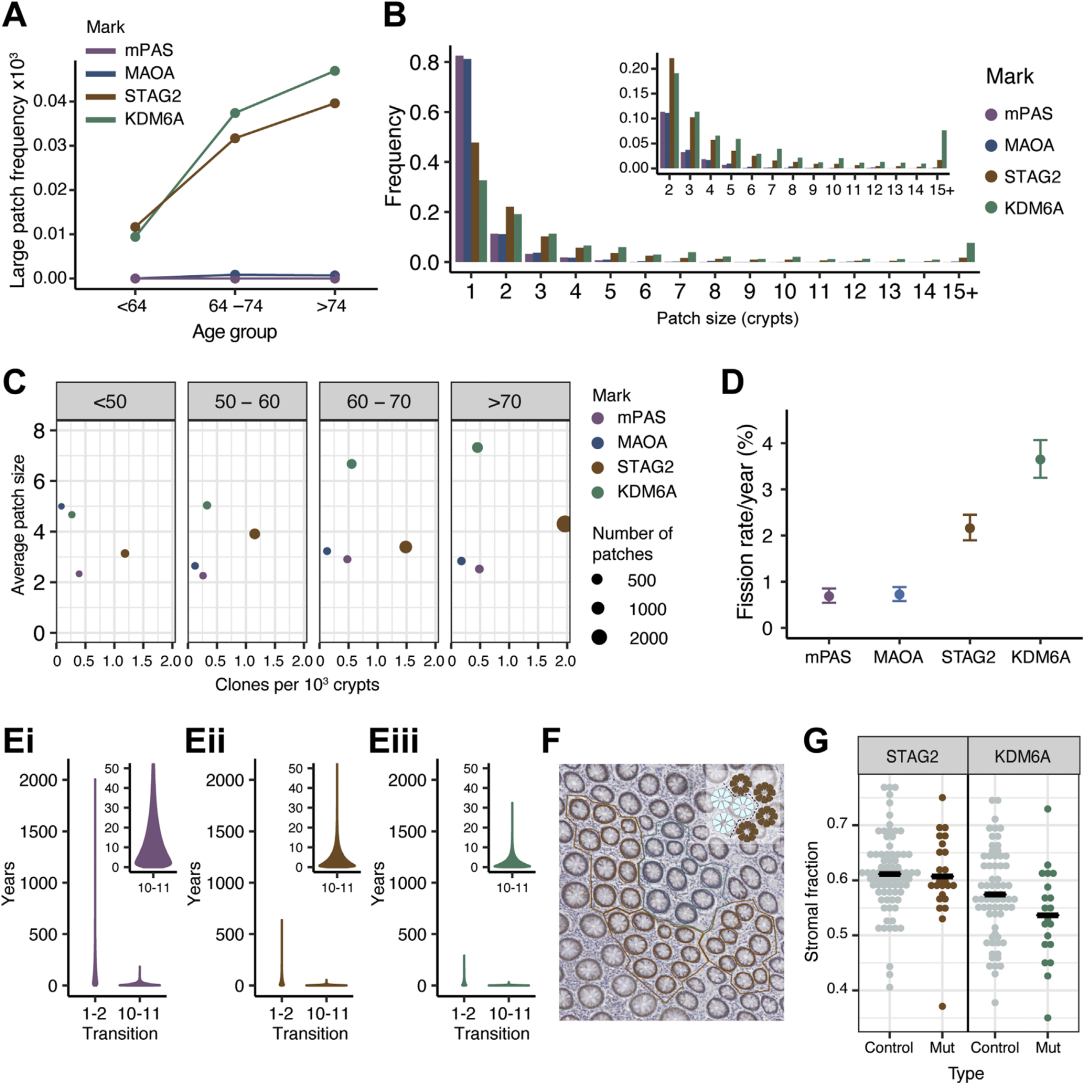
KDM6A^−^ patches lack significant overcrowding despite increased fission. (*A*) Plot showing mean frequency of large (≥10 crypts) patches for age groups shown. (*B*) Histogram showing the frequency of different patch sizes for mPAS, MAOA, KDM6A, and STAG2 across all ages. Inset shows patch size ≥2 crypts on expanded y-axis. (*C*) Dot plot of mean clone frequency plotted against mean average patch size for multicrypt clones in age groups shown for mPAS, MAOA, STAG2, and KDM6A. (*D*) Plot showing inferred fission rate/crypt/year for KDM6A compared with mPAS, MAOA, and STAG2 (data replotted from Nicholson et al^26^). Error bars = 95% CI. (*E*) Simulation data showing the time in years taken for transitions between patch sizes 1–2 and 10–11 for (*i*) mPAS, (*ii*) STAG2, and (*iii*) KDM6A. Insets show 10–11 transition on expanded y-axis. (*F*) Image showing patch selection for assessment of crypt packing density within KDM6A^−^ (*blue*) and adjacent wild-type crypts (*brown*). Inset indicates placement of borders between crypts. (*G*) Dot plots showing stromal fraction for STAG2^−^ and KDM6A^−^ and adjacent wild-type patches comprising 10 crypts.

The age-related size distribution of multicrypt clones was analyzed to infer the fission rate associated with KDM6A loss and compare it with those previously described for neutral (MAOA and mPAS) and advantaged clonal marks (STAG2).^26^ This revealed an age-related increase in the frequency of large clones (≥10 crypts/patch) for STAG2^−^ and KDM6A^−^ but not for neutral marks (Figure 2*A*). Loss of KDM6A generates a higher proportion of large patches than the other clonal marks, whereas STAG2 loss generates more clones due to a higher event rate (Figure 2*B* and *C*). From these patch size distributions, the crypt fission rate associated with loss of KDM6A was calculated to be to 3.6% per year (95% confidence interval [CI] 3.2–4.1), approximately 5-fold higher than the background homeostatic rate previously derived from neutral clonal marks (Figure 2*D*). Consequently, in individuals older than 80 years, 13.5% of KDM6A^−^ clones are found as patches comprising more than 5 crypts compared with 4.8% for STAG2, 1.7% for mPAS, and 0.8% for MAOA.

### KDM6A^−^ and STAG2^−^ Patches Lack Significant Local Overcrowding

Expanding clones are more likely to undergo additional fissions as the probability scales with the number of crypts present. Consequently, the interval between fissions decreases rapidly with increasing patch size. For example, mathematical modeling of the expansion of KDM6A^−^ crypts indicates the median time taken to grow from 1 to 2 crypts is 19 years, but only 2 years to grow from 10 to 11 (Figure 2*E*). Therefore, recently formed larger patches might be expected to demonstrate overcrowding.

**Supplementary Figure 2:**
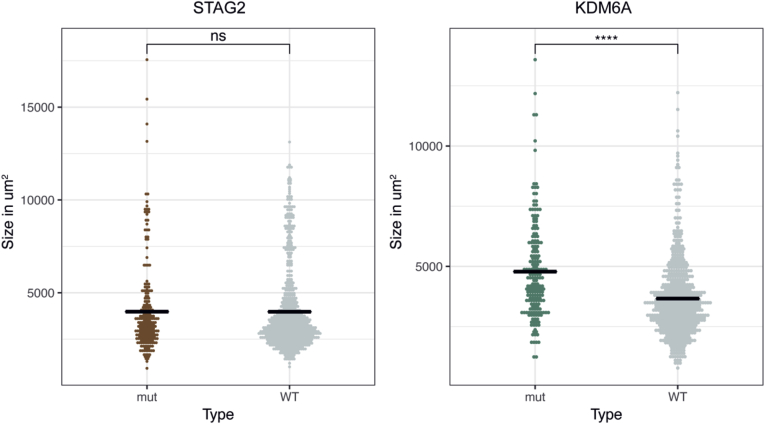
Size measurement of STAG2^−^ and KDM6A^−^ crypts. Crypt areas in multicrypt clones containing 10 crypts that are STAG2^−^ or KDM6A^−^ (24 and 20, respectively). The area of individual crypts was determined using QuPath software. As controls, adjacent or nearby wild-type groups of 10 crypts were analyzed in the same way. Jitter plots show areas of crypts. Black line = median, ∗∗∗∗*P* < .001.

**Supplementary Figure 3:**
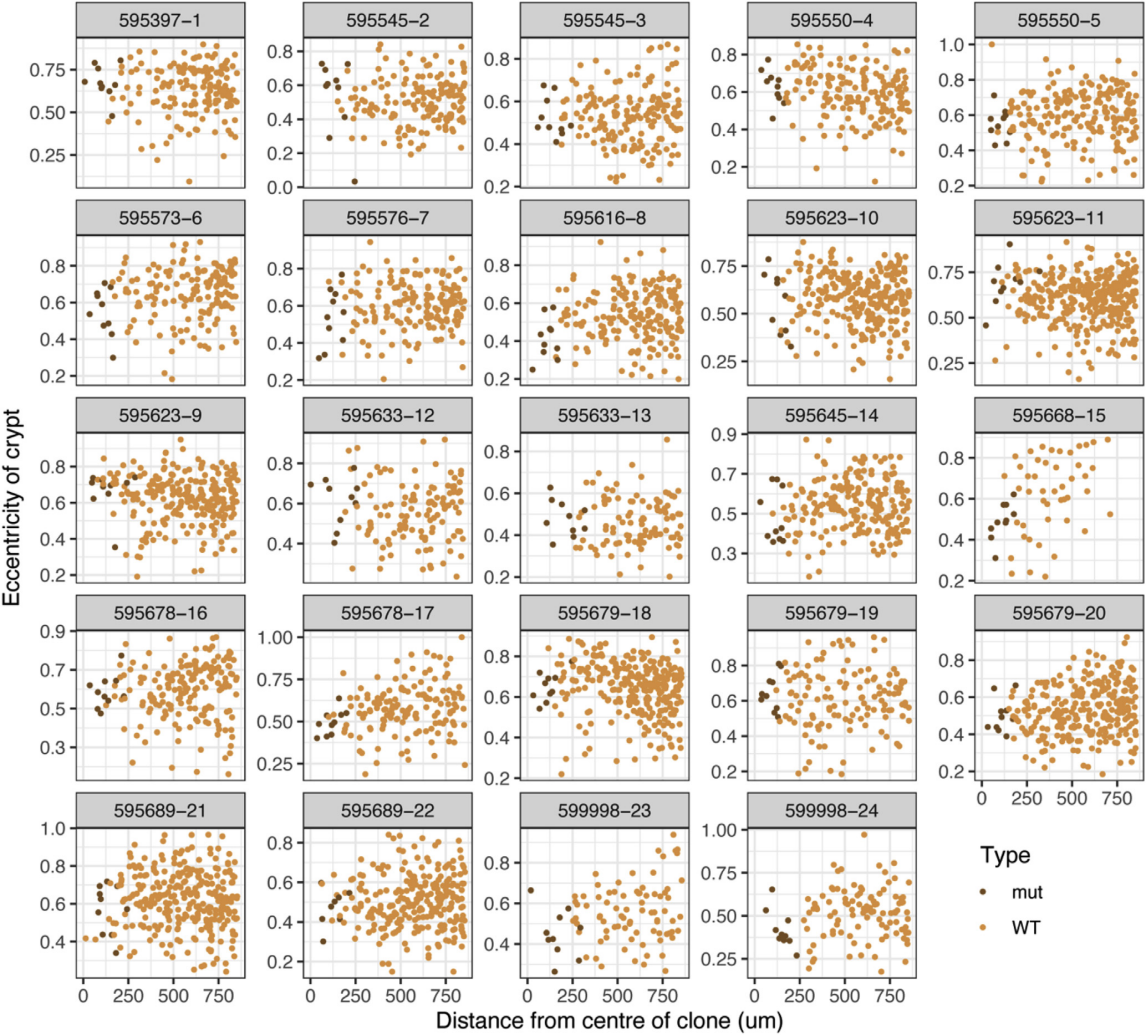
Eccentricities of crypts around STAG2^−^ clones of size 10 as a function of radial distance from crypt centroid. Crypt squashing due to clone growth would impact wild-type (WT) (*orange*) crypts closest to the expanding patch, predicting a right to left decline in the crypt eccentricities in the plots above with distance from mutant (*brown*) crypts. This pattern is not observed.

**Supplementary Figure 4:**
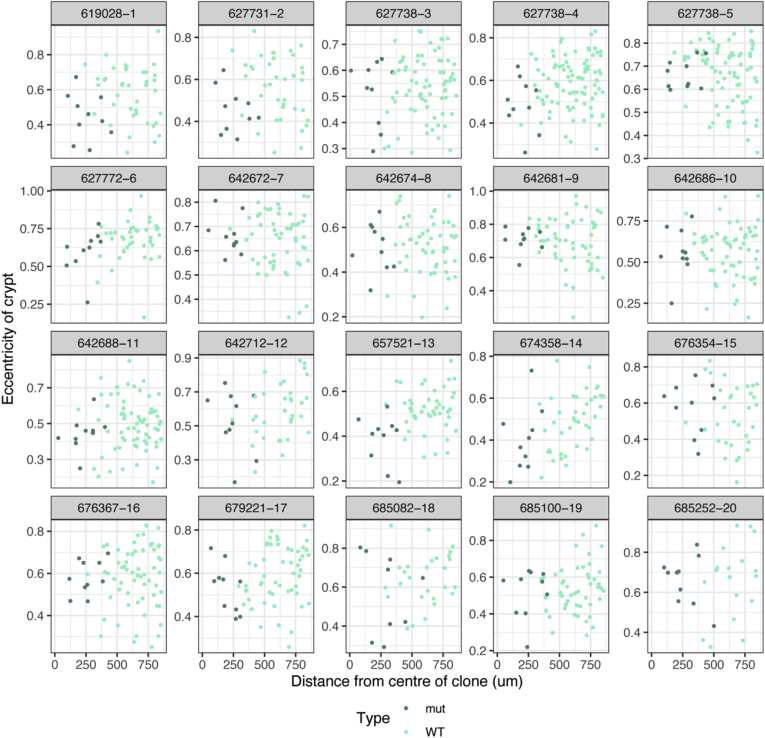
Eccentricities of crypts around KDM6A^−^ clones of size 10 as a function of radial distance from crypt centroid. Crypt squashing due to clone growth would impact wild-type (WT) (*light turquoise*) crypts closest to the expanding patch, predicting a right to left decline in the crypt eccentricities in the plots above with distance from mutant (*teal*) crypts. This pattern is not observed.

**Supplementary Figure 5:**
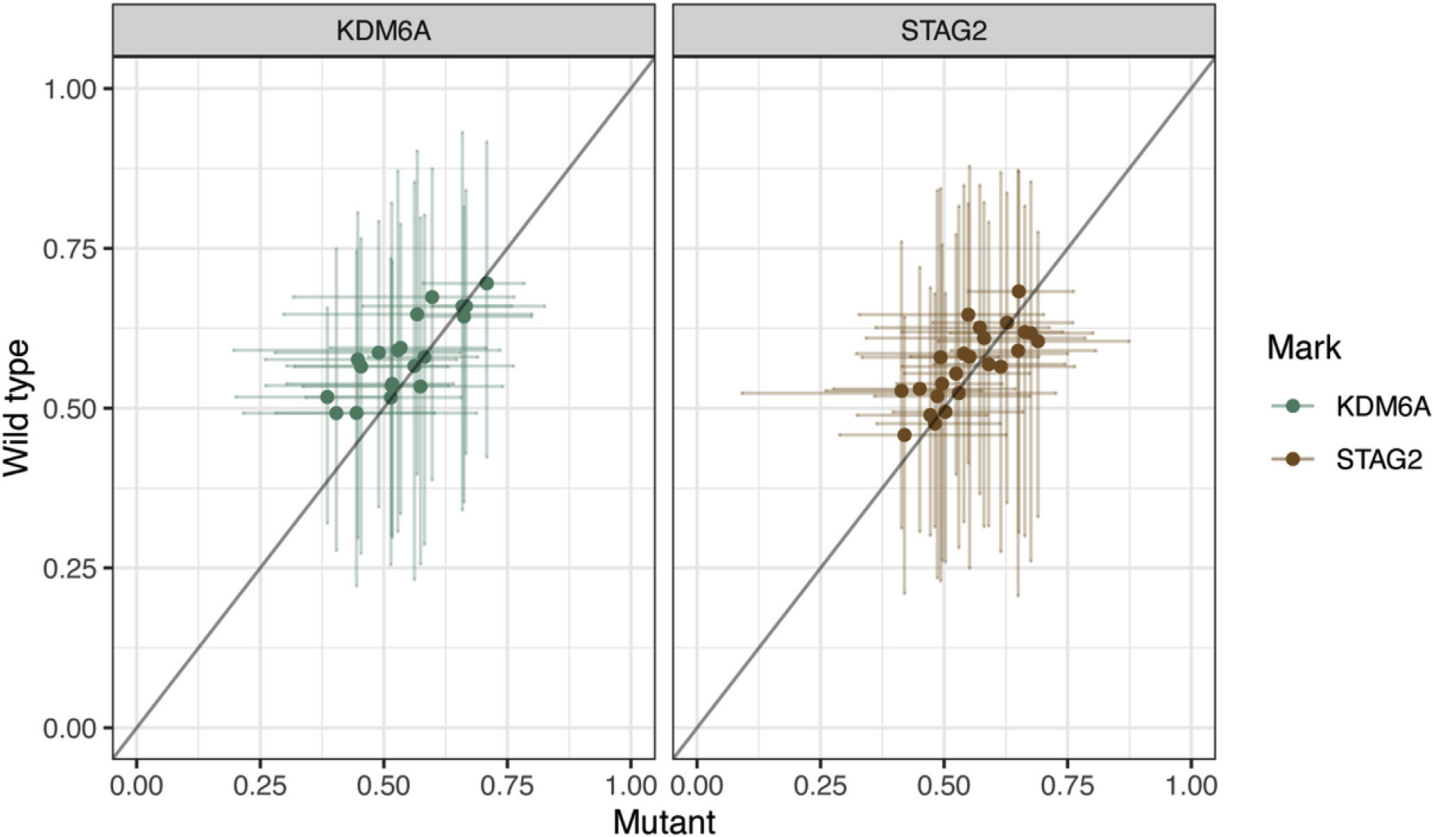
Comparison of mean eccentricities of mutant and adjacent WT crypts in clones. For each assessed patch of 10 KDM6A^−^ or STAG2^−^ crypts, the mean eccentricity of the mutant crypts is plotted on the x-axis (0 = perfect circle) against the mean eccentricity of the surrounding wild-type crypts on the y-axis. The diagonal line represents x = y, where the mean eccentricities of mutant and surrounding WT crypts correspond. Error bars = 95% CI around the mean.

To test if larger patches are more densely packed, the area occupied by crypts and their surrounding stroma was determined for 24 STAG2^−^ and 20 KDM6A^−^ clones containing 10 crypts, the largest size for which sufficient data could be obtained (Figure 2*F* and *G*). The fraction of each patch occupied by stroma was then calculated. Adjacent to each mutant clone, 3 random groups of 10 crypts were defined as control “patches” and similarly analyzed (totaling 72 control patches for STAG2 and 60 control patches for KDM6A). Comparing the fraction of each patch occupied by stroma to adjacent wild-type groupings indicated a slight trend toward increased packing density for STAG2^−^ as well as KDM6A^−^ crypts that failed to reach significance (Figure 2*G*). Considering that a lack of overcrowding may stem from a decrease in crypt size, the areas of crypts were measured. This revealed that KDM6A^−^ crypt sections are approximately 1.3 times the size of adjacent wild-type crypts (*P* < .001) (Supplementary Figure 2). No difference was found between STAG2^−^ crypts and their wild-type neighbors (Supplementary Figure 2). Therefore, lack of overcrowding cannot be attributed to reduced crypt size for either STAG2 or KDM6A loss. We also considered the possibility of accommodation of clonal expansions by “squashing” of neighboring crypts. However, analysis of crypt eccentricities provided no evidence for the predicted flattening of crypt architecture that would result (Supplementary Figure 3, Supplementary Figure 4, Supplementary Figure 5).

Together these observations suggest that crypts even within relatively recent clonal expansions avoid overcrowding to largely achieve ambient density.

### Evidence for Crypt Fusion

The lack of overcrowding in KDM6A^−^ clones suggests a mechanism counteracting the localized increase in fission. An opposing process of crypt fusion has been recognized in mouse intestine.^19^ A homeostatic human fusion rate has been estimated by assuming equivalence in the rate of both fission and fusion.^20^ On a tissue-wide basis such a balance of rates could act to maintain constant crypt density. However, local advantaged expansions can be balanced only if fission and fusion are locally coordinated.

**Figure 3:**
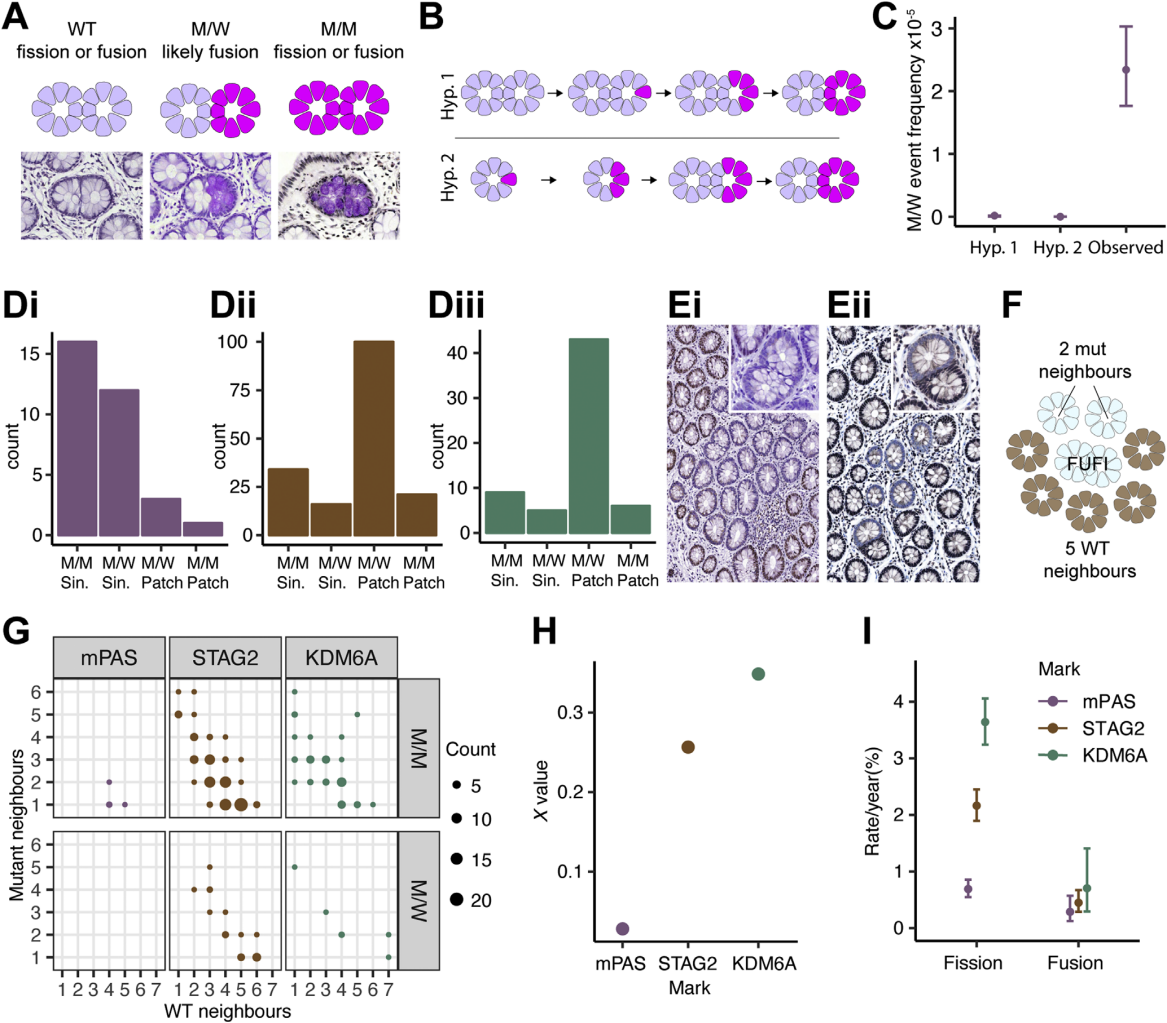
Crypt fission and fusion are independently regulated processes. (*A*) Schematic and representative mPAS-stained images of 3 types of fusion or fission forms. (*B*) Schematic representation of alternative origins of M/W forms. Hypothesis 1 (Hyp1): stem cell mutation in one branch of intermediate fission form followed by monoclonal conversion. Hypothesis 2 (Hyp 2): fission of a preexisting partially populated crypt with segregation of mutant and wild-type epithelium into each branch, followed by monoclonal conversion. (*C*) Comparison of M/W event frequencies simulated for hypotheses described in (*B*) and the observed frequency. Error bars = 95% CI. (*D*) Bar graphs showing numbers of FUFI types scored for (*i*) mPAS, (*ii*) STAG2, and (*iii*) KDM6A. (*E*) Representative images of FUFIs at patch borders: (*i*) KDM6A M/M FUFI, (*ii*) STAG2 M/W FUFI. Insets show enlarged FUFIs. (*F*) Schematic showing scoring of FUFI neighbors used for calculation of *χ*. (*G*) Dot plot showing neighboring crypt status of patch border FUFIs (M/M or M/W) for mPAS, STAG2, and KDM6A. Each *dot* represents 1 or more FUFIs for which neighbors were scored. (*H*) Plot comparing the value of *χ* for mPAS, STAG2, and KDM6A. (*I*) Plot comparing the derived fission and fusion rates for mPAS, STAG2, and KDM6A. mPAS and STAG2 fission rates correspond to data from Nicholson et al,^26^ replotted. Error bars = 95% CI.

We first sought confirmation that fusion occurs. The evidence in human epithelium is based on identification of branched crypts within which clonal loss of mitochondrial CCO activity is restricted to one branch. These are interpreted as transition intermediates in an active fusion process.^20^ Analysis of en face tissue sections stained to visualize mPAS positivity confirmed the existence of rare heterotypic branched forms in normal human colonic epithelium. Analysis of more than 2 × 10^6^ crypts in sections from 80 individuals containing mPAS^+^ clones identified 32 candidate mPAS^+^ branched forms that were either mixed (mutant and wild-type, M/W) or fully mutant (M/M) (Figure 3*A*). Of the 13 M/W forms, the positive epithelium was always restricted to 1 branch.

**Supplementary Figure 6:**
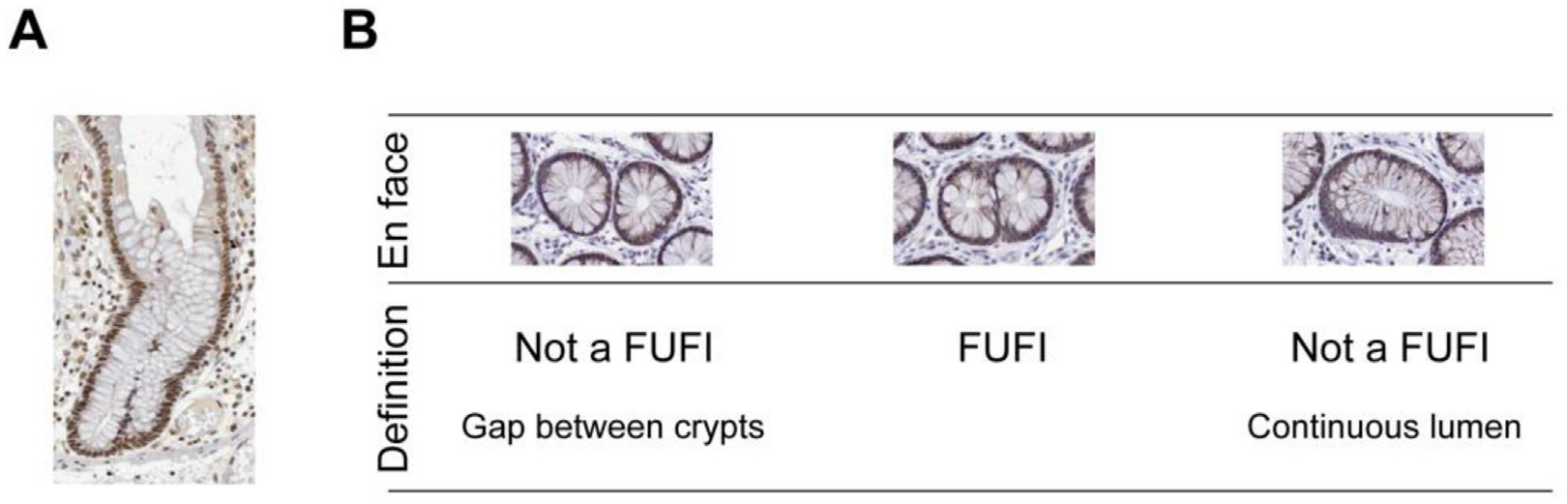
Defining the FUFI. (*A*) Representative image of KDM6A-positive bifurcating crypts sectioned longitudinally. (*B*) A FUFI is defined as 2 adjoined crypts viewed in a transverse section with 2 clearly discernible lumina lacking any separating gap.

An alternative interpretation is that branched crypts are intermediate fission forms whereby heterotypic staining arises due to mutations occurring or segregating into a single branch (Figure 3*B*). We formally considered this possibility using the fusion duration estimate derived by Baker and colleagues^20^ as well as our previous estimates of de novo mutation probability and clone fixation rates that together determine the frequency of monoclonal crypts present in individuals of different age.^26^ Within the relatively small number of branched crypts present, none are predicted to contain monoclonal crypt branches by either mechanism (Figure 3*C*). This suggests that heterotypic forms represent genuine intermediates in an active fusion process. Because the bulk of branched crypts are unstained and can represent intermediates in either fusion or fission we propose the agnostic term FUFI to describe these transition forms (Supplementary Figure 6).

### Crypt Fission and Fusion Are Regulated Independently

To calculate crypt fusion rates, heterotypic and homotypic FUFIs were also evaluated for STAG2 and KDM6A loss by scoring approximately 3.9 × 10^6^ and 1.8 × 10^6^ crypts from 53 and 102 individuals, respectively. In total, more than 28,000 FUFIs were evaluated. This identified 151 and 63 FUFIs with STAG2 (18,928 clones analyzed) and KDM6A loss (5,353 clones analyzed), respectively (Figure 3*D*). These could be found as single events or within multicrypt clones (Figure 3*E*).

Assuming equal duration for fission and fusion (ie, the time window during which FUFIs are detectable), the fusion rate is accessible by proportionality. Specifically, the ratio of the frequency of observed fission FUFIs to the fission rate (independently calculated from the patch size distribution) would equal the ratio of the frequency of observed fusion FUFIs to the fusion rate. However, although all M/W FUFIs are considered fusions, M/M FUFIs can be either fissions or fusions. Therefore, to exploit this proportionality, the relative contribution of M/M FUFI events to the total number of fusions (termed chi: *χ*) needs to be determined. The value for *χ* is calculable on the basis that M/M FUFIs have a probability of being a fusion event that depends on the number of M and W neighbors present at the onset of the fusion process. Therefore, the status of crypts neighboring FUFIs at the patch border of multicrypt clones were scored as W or M (totaling 4, 121, and 49 for mPAS, STAG2, and KDM6A, respectively) (Figure 3*F* and *G*). Single FUFIs had W neighbors only. Averaging the M and W neighbors of M/M FUFIs revealed *χ* to be 0.03 for mPAS. As neutral marks such as mPAS generate mostly small clones, most fusion events are W/M leading to low values of *χ*. At the edge of larger expansions generated by advantaged marks, M/M fusions occur more readily. Correspondingly, the *χ* values for STAG2 and KDM6A are 0.26 and 0.35, respectively (Figure 3*H*). Rates of fusion using these values of *χ* were then estimated using the proportionality described previously (see supplemental information for details).

This analysis indicates similar crypt fusion rates for mPAS, STAG2 and KDM6A of 0.3% per year (95% CI 0.1–0.6), 0.4% (95% CI 0.3–0.7), and 0.7% (95% CI 0.3–1.4) (Figure 3*I*). Comparison of mPAS fission (0.7% per year; 95% CI 0.5–0.9) and fusion rates show that these closely correspond. This suggests that in homeostasis the rates of both processes are balanced and will act together to maintain constant crypt numbers across the tissue, as has been suggested previously.^20^ However, for mutations causing elevated fission rates there appears to be no evidence for a compensatory increase of the fusion rate. These analyses suggest that fission and fusion are independent processes and not coordinately regulated.

### Crypt Diffusion Accommodates New Crypts Throughout Life

A striking feature of larger patches is that mutant crypts have over decades populated the territory initially occupied by multiple independent crypts without a significant increase in crypt density. In the absence of appreciable crypt fusion this suggests local adjustments to disperse crypts from the growing focus. With this rationale, we considered the possibility of random crypt movement in the form of a diffusion process.

**Figure 4:**
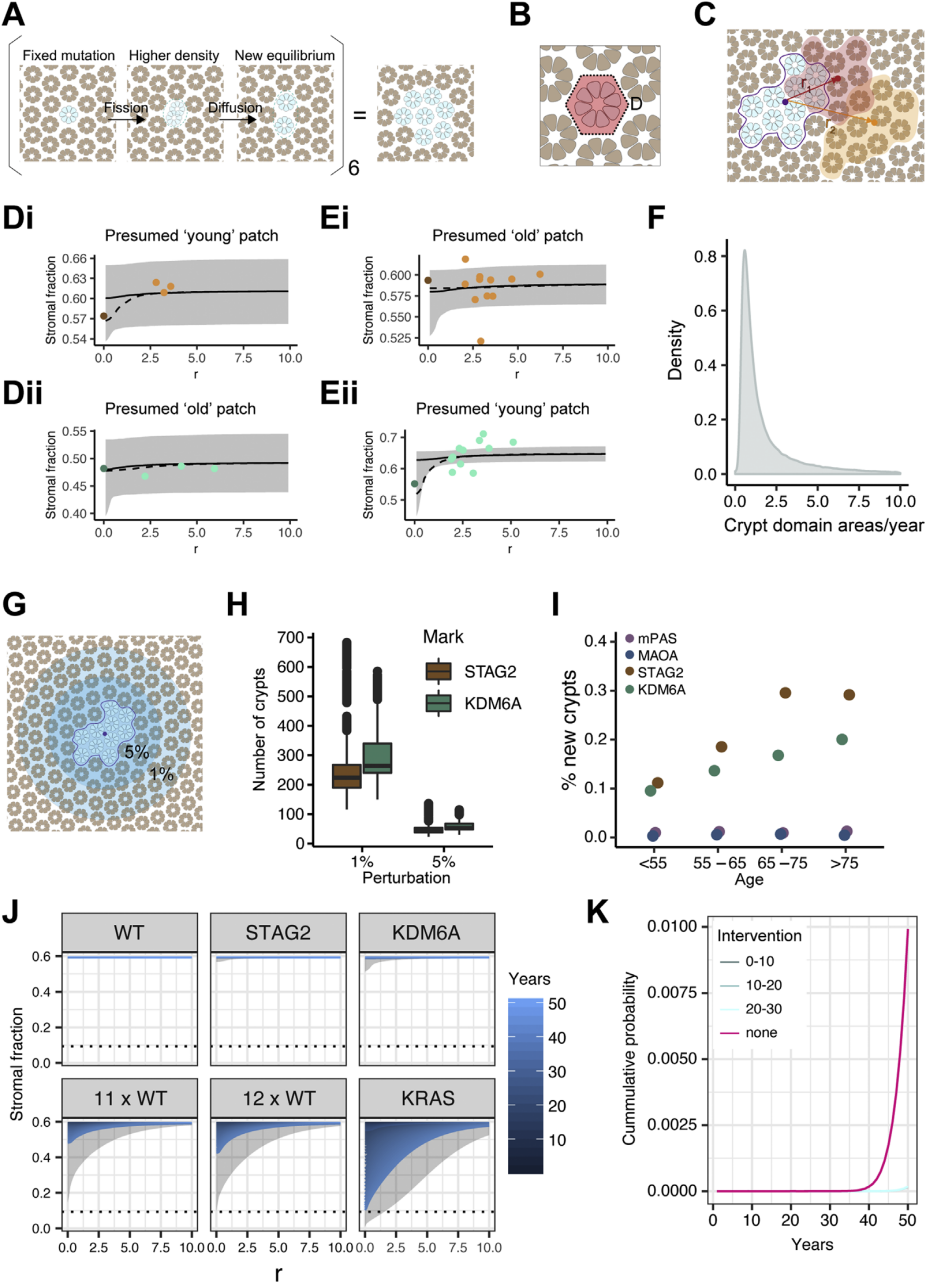
Evidence for a crypt diffusion process. (*A*) Representation of the proposed crypt diffusion process. Fission generates higher local density which is relieved by diffusion. (*B*) Schematic showing crypt cross section with area of surrounding stroma defining a crypt domain. (*C*) Representation of areas measured to assess packing of mutant patch and surrounding crypts in rolling windows placed at different distances (r) from centroid of mutant patch. (*D*) Examples of radial variation in crypt packing in patches of 10 crypts when moving from mutant clone to 3 adjacent control groupings for (*i*) STAG2 and (*ii*) KDM6A. Points are data derived, the *black line* (*gray ribbon*) is the median (95% CI) theoretical stromal fraction as fitted from the diffusion model. *Dashed line* shows the average of the 25 most likely trajectories from initial mutation to clone of size 10, based on population average diffusion and neighborhood ambient stromal fraction. (*E*) As (*D*) but with rolling window data. (*F*) Density plot of values obtained for the diffusion coefficient in human colonic epithelium. (*G*) Representation of area of crypts affected if space is decreased by 1% or 5% surrounding a mutant patch of 10. (Crypt numbers not to scale.) (*H*) Boxplot showing simulated numbers of crypts affected if spacing is decreased by 1% or 5% respectively by addition of a patch of 10 mutant crypts. (*I*) Dot plot showing median frequency of newly generated crypts for mPAS, MAOA, STAG2, and KDM6A across 4 age bins. (*J*) Line graphs showing the stromal fraction resulting from crypt diffusion and crypt fission rates corresponding to the homeostatic (WT) rate as well as those associated with STAG2, KDM6A, and KRAS and multiples of 11 and 12 of the WT rate. *Dotted line* = whitespace fraction calculated from optimal hexagonal packing of circles. r = distance from centroid of patch in crypt domains. (*K*) Line graph showing results from simulations to calculate the cumulative probability of a clone developing into a lesion, defined as reaching the whitespace fraction for hexagonal packing shown in (*J*), over time. Plots using the fission rate associated with KRAS (17 times WT, none) are shown alongside those including therapeutic crypt fission inhibition applied for a decade either immediately after mutation acquisition (0–10), after 10 years (10, 11, 12, 13, 14, 15, 16, 17, 18, 19, 20) or 20 years (20, 21, 22, 23, 24, 25, 26, 27, 28, 29, 30).

In the colonic epithelium it is the crypts that are being diffused in the “space” of the surrounding stroma (Figure 4*A*). The diffusion coefficient (change in area per unit time) can be estimated based on crypt packing (measured in terms of crypt area per unit area of mucosa) and consideration of all possible sequences of fission events, constrained by the patient age and calculated using the mutation and fission rates.

To find evidence supporting a diffusion-type process we revisited the STAG2^−^ and KDM6A^−^ patches of 10 crypts and surrounding control patches. For each mutant clone (24 for STAG2 and 20 for KDM6A) and the corresponding 3 arbitrary control groups of 10 adjacent wild-type crypts, each crypt was spatially mapped in X/Y coordinates. Total patch areas were divided in crypt domains that were defined as the area occupied by a crypt and its share of surrounding stroma (Figure 4*B*). Areas of individual crypts as well as total patch area were measured and the distance between mutant clones and wild-type patch centroids, r, was determined (Figure 4*C*).

**Supplementary Figure 7:**
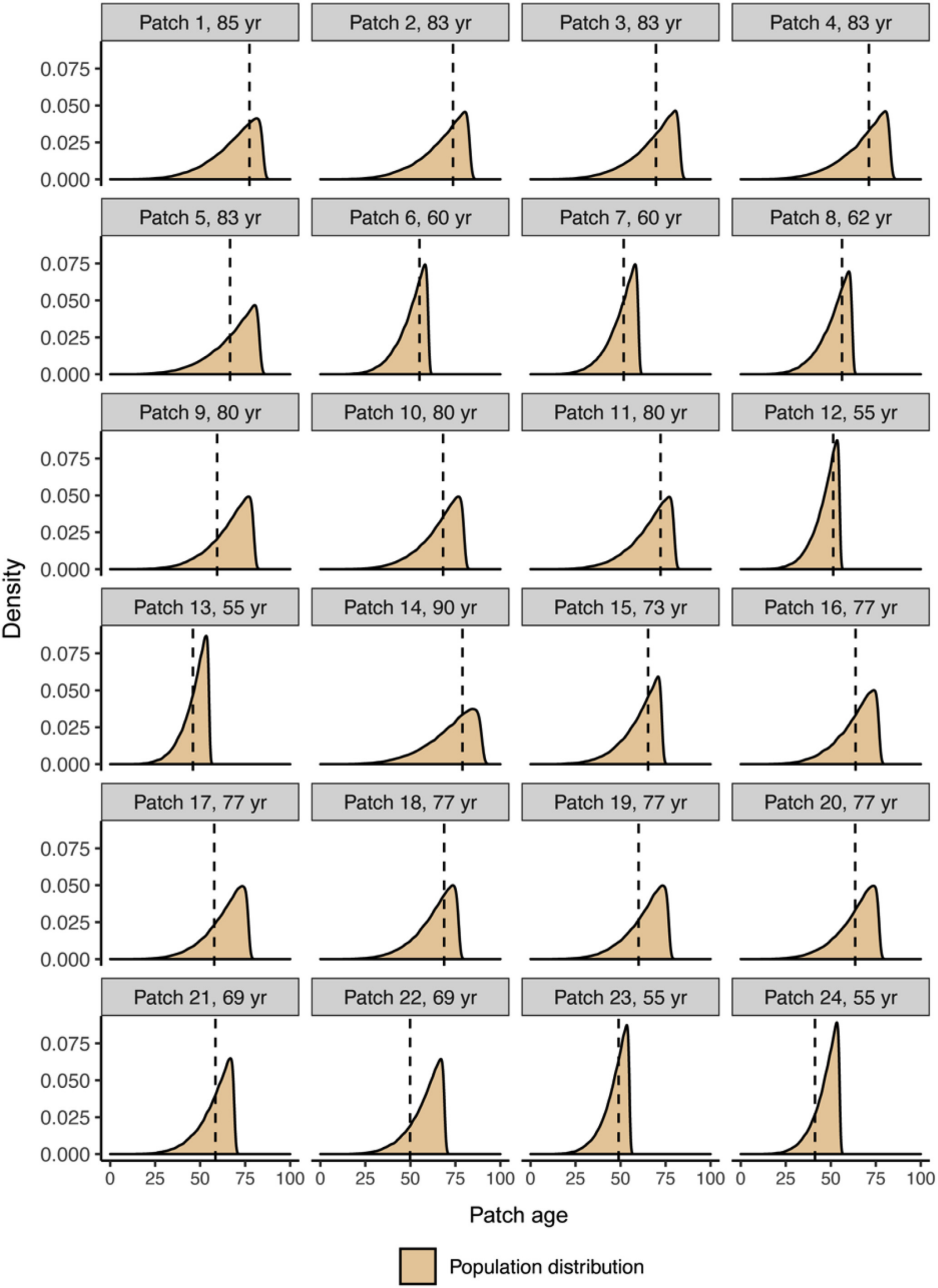
Inferred ages of STAG2^−^ clones. Theoretical distributions for the age of STAG2^−^ clones in years covering the period from single crypt to a clone comprising 10 crypts. The theoretical density of patch age is calculated using the mutation rate, the monoclonal accumulation rate, and the fission rate (modeling fission as a stochastic birth process). *Dashed lines* show the most likely patch age for each clone derived from the average of the 25 most likely trajectories.

**Supplementary Figure 8:**
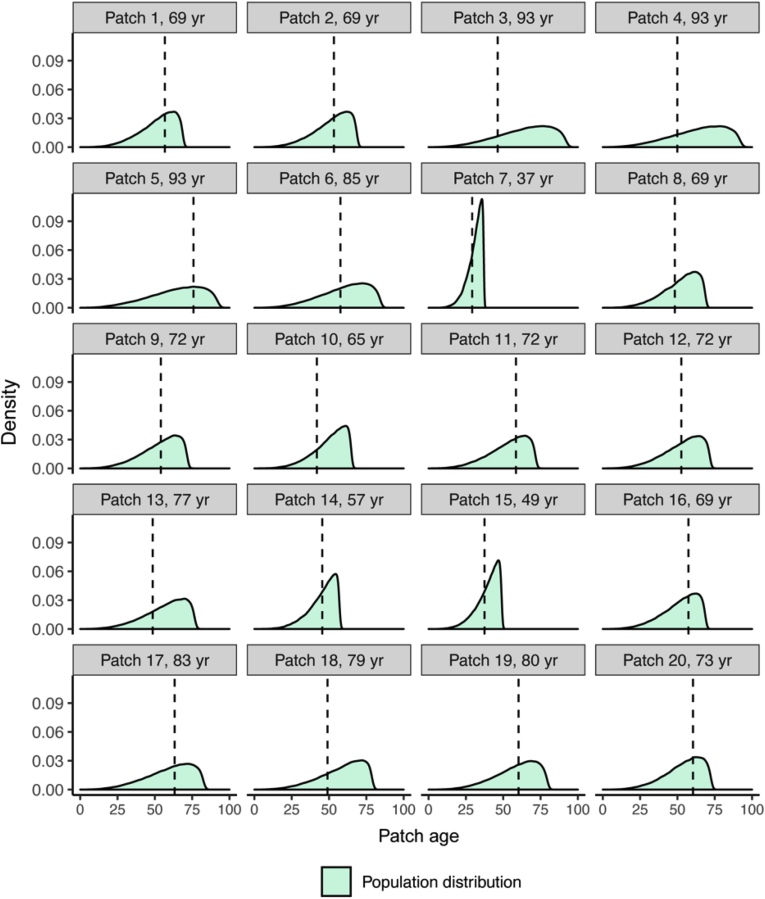
Inferred ages of KDM6A^−^ clones. Theoretical distributions for the age of KDM6A^−^ clones in years covering the period from single crypt to a clone comprising 10 crypts. The theoretical density of patch age is calculated using the mutation rate, the monoclonal accumulation rate, and the fission rate (modeling fission as a stochastic birth process). *Dashed lines* show the most likely patch age for each clone derived from the average of the 25 most likely trajectories.

**Supplementary Figure 9:**
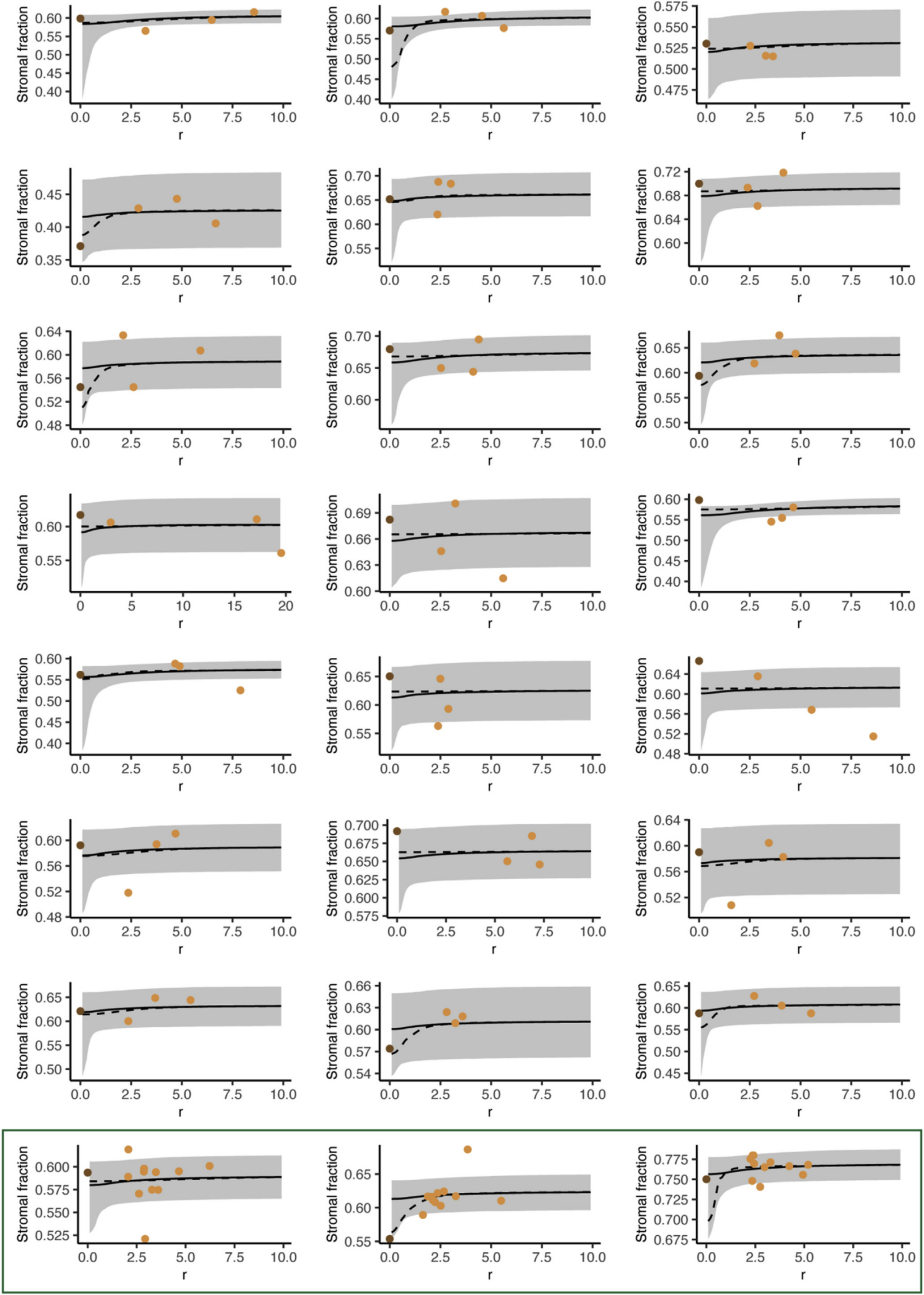
Diffusion model prediction of stromal fraction changes for STAG2^−^ clones.Plots show the stromal fraction measured for STAG2^−^ as well as surrounding wild-type (WT) patches of 10 crypts. The radial distance r (in crypt domains) is measured from adjacent patch centroid to mutant patch centroid. The *black line* and *gray ribbon* represent the median and 95% CI theoretical stromal fraction as fitted from the diffusion model. *Dashed line* shows the solution from averaging the 25 most likely trajectories from initial mutation to clone of size 10, assuming population average diffusion and neighborhood ambient stromal fraction. *Green box*: patches for which a “rolling window” was applied. For each mutant patch, surrounding measurements included 2 areas comprising 3 mutant and 7 WT, 2 mutant and 8 WT, and 1 mutant and 9 WT crypts as well as 5 surrounding WT patches at varying distances (combined: 1 data point for the mutant patch and 11 for surrounding patches).

**Supplementary Figure 10:**
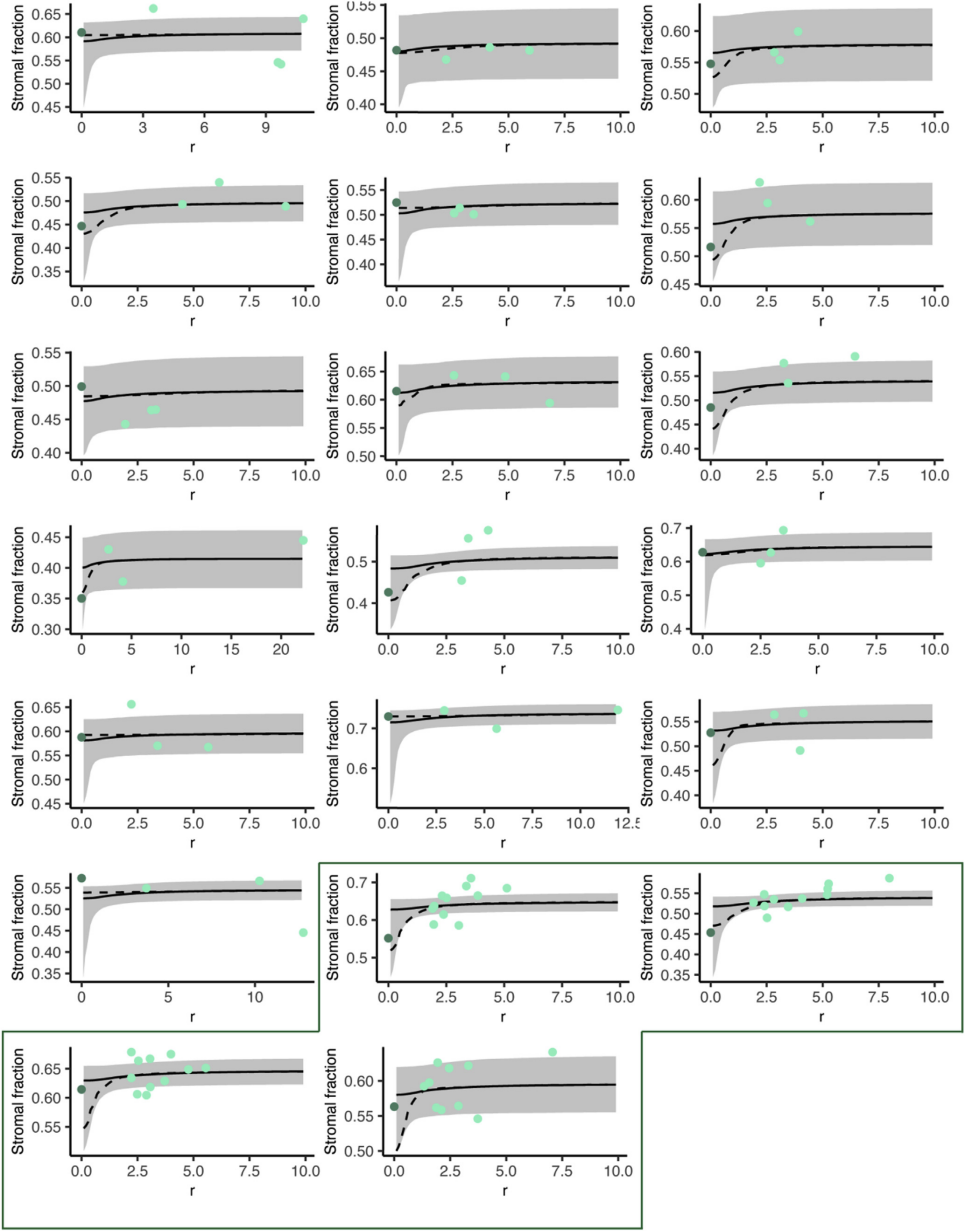
Diffusion model prediction of stromal fraction changes for KDM6A^−^ clones. Plots show the stromal fraction measured for KDM6A^−^ as well as surrounding wild-type (WT) patches of 10 crypts. The radial distance r (in crypt domains) is measured from adjacent patch centroid to mutant patch centroid. The *black line* and *gray ribbon* represent the median and 95% CI theoretical stromal fraction as fitted from the diffusion model. *Dashed line* shows the solution from averaging the 25 most likely trajectories from initial mutation to clone of size 10, assuming population average diffusion and neighborhood ambient stromal fraction. *Green box*: patches for which a “rolling window” was applied. For each mutant patch, surrounding measurements included 2 areas comprising 3 mutant and 7 WT, 2 mutant and 8 WT, and 1 mutant and 9 WT crypts as well as 5 surrounding WT patches at varying distances (combined: 1 data point for the mutant patch and 11 for surrounding patches).

Using known mutation and fission rates, the potential trajectories from initial mutation to 10-crypt mutant clones were simulated and the most likely trajectories were used to calculate the age of the clone (Supplementary Figures 7 and 8). The diffusion coefficient was inferred to define an overall tissue process that best fits the differences in stromal densities between mutant and control patches. It describes how the burden of decreased stromal fraction resulting from a mutant clone is dispersed into the surrounding tissue over time. For older clones, the expectation is for the system to be close to the ambient density, whereas for younger clones, the perturbation to the local stromal fraction may still be evident. Examples of both presumptive young and old clones were readily detectable (Figure 4*D*, Supplementary Figures 9 and 10).

**Supplementary Figure 11:**
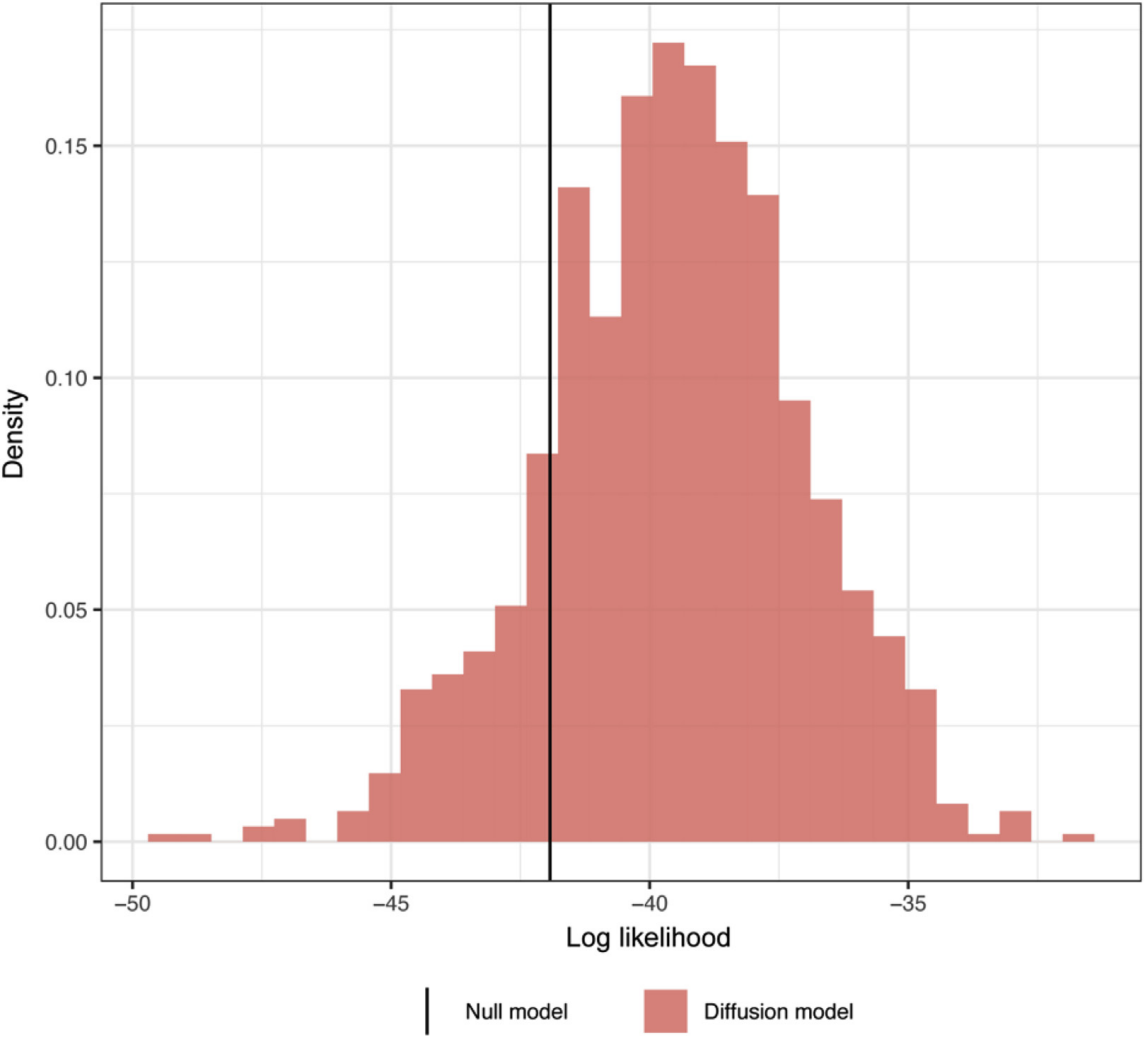
Comparison of diffusion model to null model. Results for model fit comparison where the log likelihood of the data under a null model with constant per-clone stromal fraction is compared with the log likelihood of the data under the diffusion model for 1000 potential trajectories (from wild-type to 10-crypt mutant clone) per clone. The *black line* lies to the left of the density mass, meaning there is evidence for a diffusion-like radial dependence in the crypt packing data.

For a subset of 7 patches (4 for KDM6A, 3 for STAG2), a more detailed rolling window analysis was performed in which the preceding approach was applied but where fields of 10 crypts were moved outward from the mutant clone. Again, evidence of a reduction in stromal fraction consistent with perturbation in younger clones was observed (Figure 4*E*, Supplementary Figures 9 and 10). The diffusion coefficient was found to be 1.05 crypt domain areas per year (95% CI 0.339–9.70) (Figure 4*F*). Reassuringly, testing a null hypothesis, that there is no diffusion-type process and therefore no radial dependence in crypt packing, by considering neighborhood ambient densities of crypts across all patches (mutant, wild-type, or mixed), generated a significantly worse fit than the experimental comparisons (Supplementary Figure 11). Of note, this confirmation of radial dependence in packing argues against other possible alleviators of crypt density such as changes in the size or shape of crypts within mutant clones.

The inferred diffusion process can be used to define the number of crypt domains impacted to accommodate a new clonal expansion. For example, the model suggests that patches of 10 KDM6A^−^ crypts would require 264 crypt domains to undergo a 1% reduction in their spacing, whereas a 5% reduction would only require 53 crypt domains (Figure 4*G* and *H*).

### Defining a Homeostatic Threshold

Limited evidence suggests that there are no significant age-related changes in colon length and crypt density.^18^ With respect just to STAG2 and KDM6A mutations, the relatively few new crypts arising during life could be easily accommodated by crypt movement. By the time individuals exceed 75 years of age, for every 10^5^ crypts, fission has added only approximately 200 and 290 new STAG2^−^ and KDM6A^−^ crypts, respectively (Figure 4*I*). However, it seems highly probable that additional genetic variants will also promote fission to different degrees. The potential for diffusion to locally balance this process as fission rate increases was investigated.

**Supplementary Figure 12:**
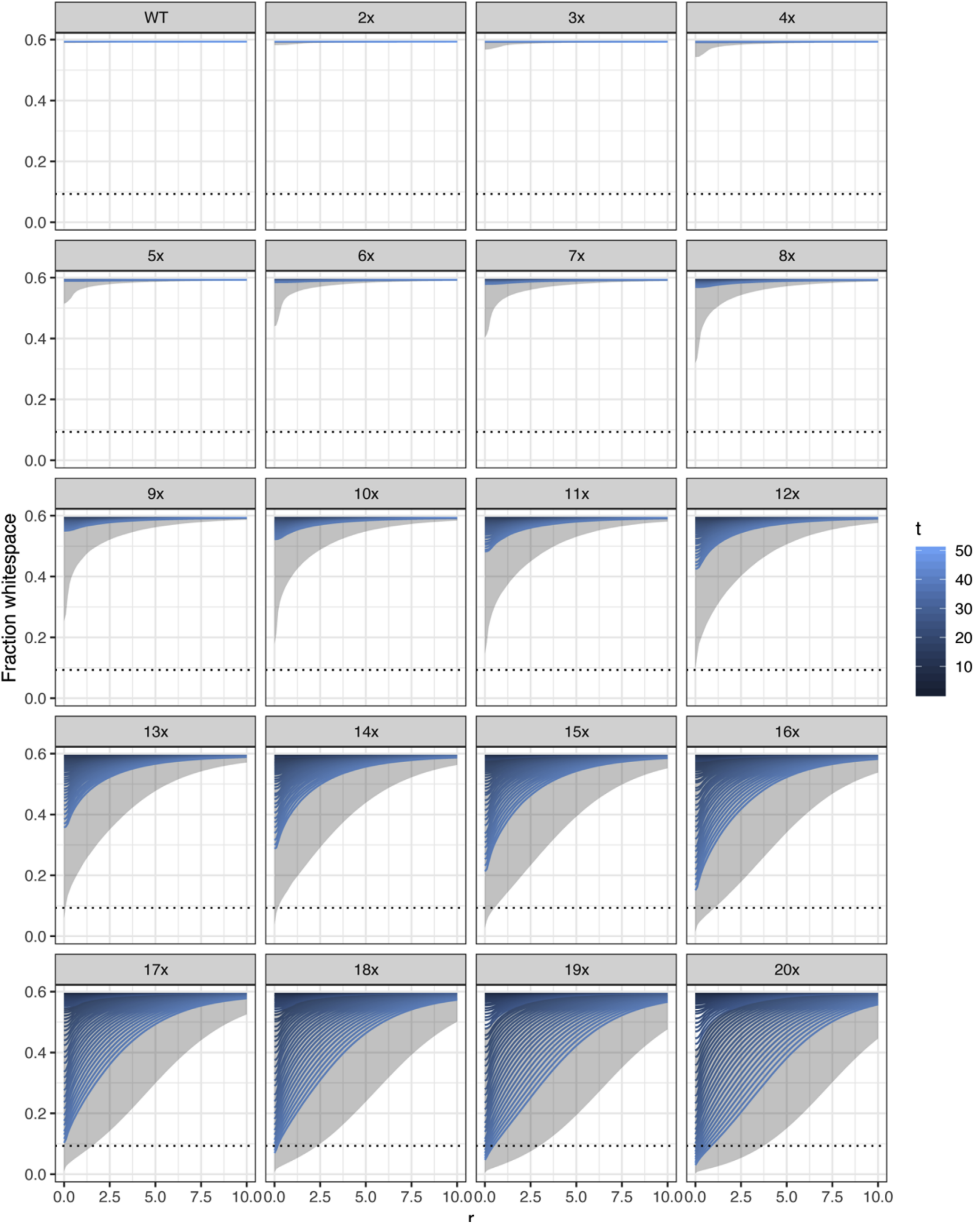
Simulating the “breaking point.” Simulations were performed to find the fission rate at which clones may generate new crypts more quickly than can be accommodated by crypt diffusion. Line graphs show the stromal fraction resulting from crypt diffusion and different crypt fission rates in multiples of the homeostatic (wild-type) rate (0.7% per year). *Dotted line* = stromal fraction calculated from optimal hexagonal packing of circles. r = distance from centroid of patch in crypt domains. *Gray area* = 95% CI.

**Supplementary Figure 13:**
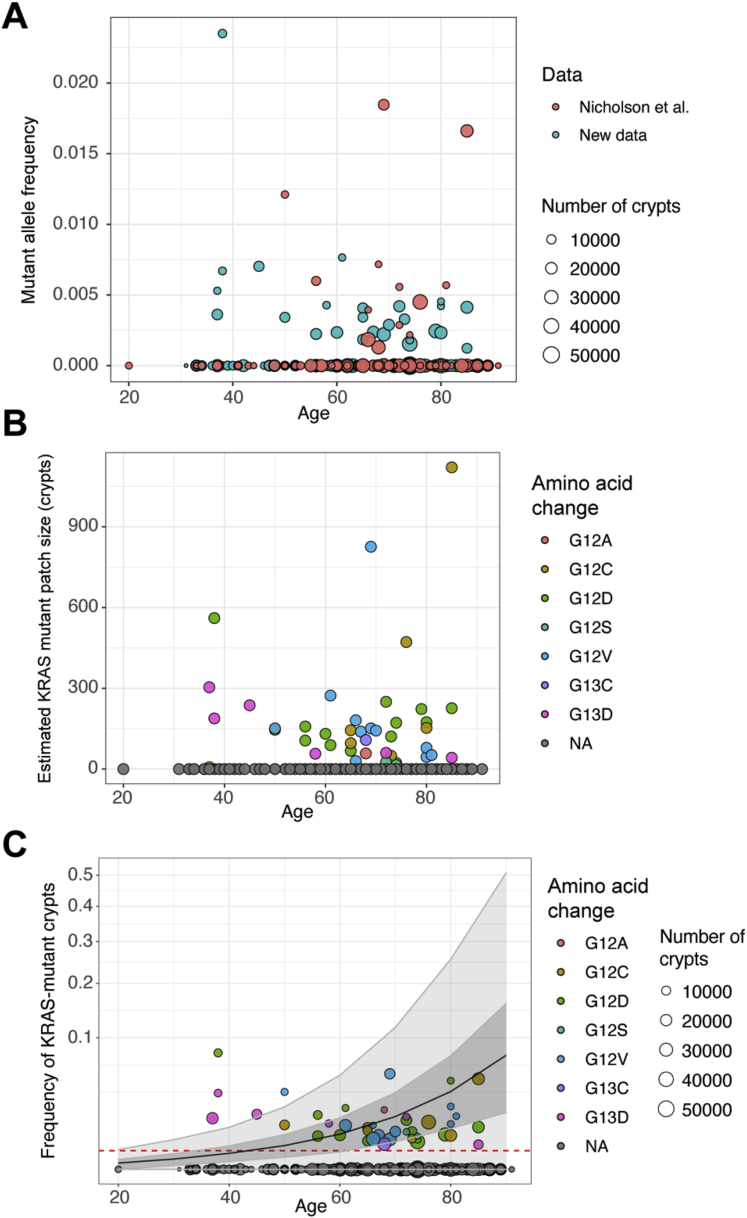
Evidence for expansion of *KRAS*-mutant clones in human colon. Targeted amplicon sequencing focusing on *KRAS* codons 12 and 13 was performed on DNA from FFPE tissue sections from 256 patients of the age range 20 to 91 years. In total, 35 individuals displayed detectable mutations. (*A*) Mutant allele frequency data of *KRAS* codons 12 and 13 mutations plotted against age. Data previously published separated from new data obtained as part of this study. (*B*) *KRAS*-mutant patch sizes inferred from the mutant allele frequencies and known crypt numbers for 256 individuals plotted against age. (*C*) Frequency of *KRAS* mutant crypts for 256 individuals plotted against age. The mean accumulation of mutant clones using the model is plotted in *black* as well as the 95% CI in *gray*. *Red dotted line* shows detection threshold.

Simulations were performed escalating the homeostatic fission rate of 0.7% per year (Supplementary Figure 12). When fission rates remain below approximately 12-fold that of homeostasis, diffusion can generate enough space to accommodate newly generated crypts (Figure 4*J*). Higher fission rates result in a proportion of clones reaching a threshold of maximum packing density within which crypts are directly touching. This suggests a potential boundary for polyp growth that is dependent on the physical processes of fission and diffusion. We have previously used targeted sequencing of FFPE sections to infer the effect of KRAS activating mutations on crypt fission.^26^ Here, in expanding on that initial dataset (see Methods) *KRAS* activating mutations were found in 35 (22 new) of 256 individuals (130 new) in the age range 20 to 91 years, corresponding to 13.7% of the cohort (Supplementary Figure 13). Mutant allele frequencies in the range of 0.12% to 2.35% combined with total crypt numbers per section enabled estimation of clone size (Supplementary Figure 13). Subsequent mathematical inference indicates that a 17-fold increase in crypt fission rate to 12% (95% CI 10.8–13.7) per year best fits with the data. Approximately 1% of *KRAS* activating mutations are predicted to breach the threshold for lesion growth after 50 years (Figure 4*J*). A therapeutic intervention inhibiting crypt fission for any 10 years could reduce this to approximately 0.01% (Figure 4*K*).

## Discussion

Several studies have identified large mutant expansions in seemingly normal epithelia.^1^^,^^2^^,^^26^ In the adult colon, this occurs by increased crypt fission rate, whereby biased mutations can generate large clones that are appropriately distributed within the tissue.^26^ In contrast, elevated glandular fission rates are also known to drive the overgrowth of adenomas and CRCs, suggesting that differences in the rate of fission or the response to it must differ between normal and neoplastic tissues.^4^, ^5^, ^6^, ^7^

In considering the epithelial responses that compensate for elevated fission rates, we first validated a new advantaged clonal mark, KDM6A, that together with STAG2 provided gene-specific assays with 5- and 3-fold increased fission rates, respectively. Comparing the configuration of the size and frequency of clones for both genes demonstrates the different strategies by which age-related mutational burden can be achieved. STAG2 has the higher mutation rate and generates many relatively small clones, whereas KDM6A generates fewer but larger expansions. A corollary of the exponential growth of patches as their size increases is that larger patches will tend to be the most recent and therefore most likely to contain evidence of local adaptation to accommodate new crypts.

The recently recognized process of crypt fusion offers a potential mechanism to compensate for fission.^19^ Occurring at equal rates in homeostasis they could effectively balance crypt numbers on a population basis.^20^ In considering fusion as a mechanism to accommodate new crypts, a baseline estimate was first established here and found to approximate that for fission; however, no upregulation of fusion accompanying the local expansions resulting from STAG2 and KDM6A mutation was identified. Conceivably other mutations may impact fusion to ease local packing but it does not appear necessary to do so.

Multicrypt clones that form over decades populate the territory previously occupied by multiple independent crypts. Aiming to understand this dispersal, we sought and found evidence of a diffusion-type process in a subset of clones. These are consistent with a recent expansion “caught in the act” of being restored to an ambient crypt density. The behavior captured probably reflects a passive dispersal mechanism rather than actual diffusion and must be accompanied by some level of stromal turnover.

The diffusion coefficient defines the rate of movement of crypt domains and the size of the larger impacted zone that is required to absorb new crypts. Parameterizing the process allows testing of the robustness of the tissue to deal with localized accelerated growth conferred by biased mutations. From this analysis, the homeostatic dispersal mechanism seems able to accommodate increased fission rates of more than 10-fold above baseline. Even for mutations that generate higher crypt fission rates, only the fastest growing clones would overgrow the available space. For example, approximately 5% of clones carrying a gene mutation that confers a 19-fold increase in fission rate would reach a threshold where they lack a stromal domain between crypts and overgrow the available space after 50 years.

The actual threshold at which clonal expansions become recognizable as pathologies may be lower than the extreme one applied here. However, the implication remains that the distinction between phenotypically normal clones and those forming overt pathologies may be determined solely by a probabilistic process in which a recent succession of fission events overwhelms homeostatic dispersal mechanisms.

Activating mutations of KRAS have been described in normal colonic epithelium. The revised estimate of a 17-fold increase for the fission rate conferred by KRAS activating mutations is higher than that previously inferred (10-fold) and is based on analysis of many more patients.^26^ It is intriguing that activating mutations of KRAS breach the extreme threshold defined here. KRAS is commonly mutated in a broad spectrum of benign and premalignant pathologies such as serrated lesions and adenomas that may arise at least in part due to the dispersal threshold being reached.^29^, ^30^, ^31^, ^32^, ^33^

These findings have clinical significance with respect to bowel cancer screening programs with the implication that clonal expansions with a high malignant potential are not all contained within visible lesions such as sessile serrated adenomas despite having a comparable tissue footprint. Further, it is known that a proportion of adenomas spontaneously regress when observed in longitudinal studies.^34^, ^35^, ^36^ One plausible explanation for such phenomena is that these lesions first develop due to reaching the threshold resulting in local overcrowding but are transient because of ongoing crypt dispersal.

Loss of function mutations affecting the APC tumor suppressor gene are also initiated and expanded by glandular fission.^4^, ^5^, ^6^, ^7^ Mutation of both APC and KRAS is frequent in CRCs. The 2 pathways are known to interact at the molecular level.^37^ It is likely their combined activation will also synergize to further elevate gland fission rate and promote overgrowth as fully neoplastic CRCs develop.

Obesity, a known risk factor for CRC, is known to be accompanied by increased crypt fission rate.^38^ Furthermore, diets deficient for methyl donors are known to reduce crypt fission rates in the mouse.^39^ An implication of the colon having the capacity to absorb many more new crypts is that modest time limited reductions in fission rates may not only slow the growth of lesions but prevent them from forming at all.

## Figures and Tables

**Supplementary Table 1:** Primary Antibodies Used for Immunohistochemistry

Antigen	Antibody	Supplier	Titer
KDM6A	HPA002111	HPA	1:100
KDM6A	#33510	CST	1:200
MAOA	SC-271123	Santa Cruz	1:200
STAG2	LS-B11284	LSBio	1:1000

**Supplementary Table 2:** Primers Used for Amplification of FFPE to Assess Amplifiability

Primer	Sequence (5' – > 3')
MAOA_150_F1	ACCCATCAGTTACTCCTTCCC
MAOA_150_R1	GGGATTAAAGCTGGGAGTTTCT
MAOA_150_F2	TAGCAGGGCCTTGAATCTGT
MAOA_150_R2	GATAGTGCCCAGAGTCACCA
STAG2_150_F1	GGAGAAGAAGACACAGTTGGATG
STAG2_150_R1	TTCTGTGAGGCATTTAGGGAAAA
STAG2_150_F2	CCTATGCTCGCACAACTATGAG
STAG2_150_R2	GGAAGCCACACATCCTCTCT
CASD1_150_F1	ACCTGGAAACCCTATGCTCAA
CASD1_150_R1	TGCAGCTATACATGCCAACC
LOC_150_F1	TCGTCTGCTTCATCCTCCTC
LOC_150_R1	GCCTAACATGCTTGGACCAC
MAOA_250_F1	TGCAAGTCTTAGGTTGGTTGC
MAOA_250_R1	TCAGTAATGGGTCATGTGCAAA
MAOA_250_F2	AAGACATGTAGGGTTGGGGC
MAOA_250_R2	CAGAACACCCTGCTCTAACCT
STAG2_250_F1	GACTCTAAGGCCAGGTCAGG
STAG2_250_R1	GGAGGTGAGTTGTGGTGTCT
STAG2_250_F2	GCCTAATCATTCTCCCTGACCT
STAG2_250_R2	TGGTGTCAAAATCCATTCCCTC
CASD1_250_F1	GGTTAGAGGAAGACAAAAGTGGA
CASD1_250_R1	CCTCAGTCCACACTTTGATACAC
LOC_250_F1	AGCTTACCTCTTTGTCTCTTCCT
LOC_250_R1	CAACCTCAAAGTATCACGTGGA
MAOA_350_F1	TTCCTTCAGAAATTGAATCCTTG
MAOA_350_R1	CCTGGGAGAAAGCAAAATCA
MAOA_350_F2	TCCCGGAGTATCAGCAAAAG
MAOA_350_R2	CATGAGAGACCCCCAAACAC
STAG2_350_F1	TCCGAATATTTTTGGTGCATT
STAG2_350_R1	CAGAGCCTTGATGAGTGCTG
STAG2_350_F2	TCTGAAGGAATGCTATGGTATGAA
STAG2_350_R2	TTGTCAAGGGTCATAGACACAA
CASD1_350_F1	CTTTGGGAAGCTTTGCGTAAAA
CASD1_350_R1	CGATTCAGGAAGATGTAAGCCA
LOC_350_F1	TCAGGAATGATGGTCTACGTGA
LOC_350_R1	TCTCAGCTCTATTCCGTGAGT

**Supplementary Table 3:** Primers Used for Amplification of KDM6A

Primer	Sequence (5' – > 3')
1_F	ACACTGACGACATGGTTCTACACGCTTTCGGTGATGAGGAAA
1_R	TACGGTAGCAGAGACTTGGTCTCCGTACCTGTCCAGTCCG
2_F	ACACTGACGACATGGTTCTACATCTTTCAGGGCAATTAAAGCATT
2_R	TACGGTAGCAGAGACTTGGTCTACAACCTACCTTTAAACTAGACTCA
3_F	ACACTGACGACATGGTTCTACAGTACAATTGGACCATGGCCA
3_R	TACGGTAGCAGAGACTTGGTCTAGTGCAGAGGTATTACTACAACTT
4_F	ACACTGACGACATGGTTCTACACAGGATGCCATTAAATGCTACTT
4_R	TACGGTAGCAGAGACTTGGTCTTCTGGGGAAATATGTGGCTTT
5_F	ACACTGACGACATGGTTCTACAATGCTGTGTCACATCCTCCA
5_R	TACGGTAGCAGAGACTTGGTCTACTTGTTTGCTACCTCTACTCCT
6_F	ACACTGACGACATGGTTCTACATGACAGATGAGACCAACAGGA
6_R	TACGGTAGCAGAGACTTGGTCTCAGGCTGAGAGACGCTAGG
7_F	ACACTGACGACATGGTTCTACACTGCCTACAAACTCAGTCTCTG
7_R	TACGGTAGCAGAGACTTGGTCTCAGAAAAGGGTCCATTGGCC
8_F	ACACTGACGACATGGTTCTACATAACCGCACAAACCTGACCA
8_R	TACGGTAGCAGAGACTTGGTCTTCTCTCAAAGTGTATAAAACCCAGT
9_F	ACACTGACGACATGGTTCTACACGACCTCTCTCTTCCACTGG
9_R	TACGGTAGCAGAGACTTGGTCTAATGCCTTGTTGTCCACCTG
10_F	ACACTGACGACATGGTTCTACAGGCTGCTCTCAATCACCTCT
10_R	TACGGTAGCAGAGACTTGGTCTGCAGTGCTGTTAGGTGTCTC
11_F	ACACTGACGACATGGTTCTACAGAGACACCTAACAGCACTGC
11_R	TACGGTAGCAGAGACTTGGTCTTCCCATCAACAAGGCAGAGA
12_F	ACACTGACGACATGGTTCTACAGCCATTTCAACAGCAACACC
12_R	TACGGTAGCAGAGACTTGGTCTGGGGCTCTGAGATTCTTCCA
13_F	ACACTGACGACATGGTTCTACAGGAAGAATCTCAGAGCCCCA
13_R	TACGGTAGCAGAGACTTGGTCTCACACTAACCTGCATGCCTT
14_F	ACACTGACGACATGGTTCTACAATGGACTTGTGCAAATGCCTAGTAA
14_R	TACGGTAGCAGAGACTTGGTCTTGGAGGTGGACATTTATCCAACAA
15_F	ACACTGACGACATGGTTCTACATGTTTTCCTGAGATCTAACCACA
15_R	TACGGTAGCAGAGACTTGGTCTCAAGGCCACGTATTACTGTAACA
16_F	ACACTGACGACATGGTTCTACATGTAGAACACTAAACTAGACTGCT
16_R	TACGGTAGCAGAGACTTGGTCTACACAGTATTAGAAACATGCCTTTT
17_F	ACACTGACGACATGGTTCTACAGTTCTGGGAGGAGGAGGAAA
17_R	TACGGTAGCAGAGACTTGGTCTAGCACAGGATAACTCTTTGCA
18_F	ACACTGACGACATGGTTCTACAAAACTTCCACAGGTATTTGTAGC
18_R	TACGGTAGCAGAGACTTGGTCTCCAACATGGCTTAGAAGATTTCC
19_F	ACACTGACGACATGGTTCTACAGTGGAAGTTGCAGCTACATGA
19_R	TACGGTAGCAGAGACTTGGTCTTGCTCCCTGGAACTTTCATG
20_F	ACACTGACGACATGGTTCTACAACCGTGTGCTAACCAATTGC
20_R	TACGGTAGCAGAGACTTGGTCTACAAACCATTCACAGTCACCT
21_F	ACACTGACGACATGGTTCTACAGGAGCTTCTTAATGTAGTTGATCC
21_R	TACGGTAGCAGAGACTTGGTCTGCTGAATAAACCTATACACTGGAAC
22_F	ACACTGACGACATGGTTCTACACTAATGGGTTCTTGGTGGCC
22_R	TACGGTAGCAGAGACTTGGTCTTGAACCCAATGAACAGTGCC
23_F	ACACTGACGACATGGTTCTACAGCTGGTCACAAATAATTTCTCCC
23_R	TACGGTAGCAGAGACTTGGTCTTGAGCTGGTTCTTCTTTTGTCC
24_F	ACACTGACGACATGGTTCTACAACTTGGAAAACTTTGTGGTGCT
24_R	TACGGTAGCAGAGACTTGGTCTCACTGCTGCTTCATAACCCA

F, forward primer; Number, amplicon number; R, reverse primer.

NOTE. Sequence includes Fluidigm CS adapters.

**Supplementary Table 4:** Primers Used for Amplification of Area Around KRAS Codons 12 and 13 as Well as the Mimic Gene PITPNM2

Primer	Sequence (5' – > 3')
KRAS_1_F	ACACTGACGACATGGTTCTACATAAGGCCTGCTGAAAATGACT
KRAS_1_R	TACGGTAGCAGAGACTTGGTCTATGGTCCTGCACCAGTAATATG
MIMIC_1_F	ACACTGACGACATGGTTCTACATAGCACCCAGCCAGCTTG
MIMIC_1_R	TACGGTAGCAGAGACTTGGTCTATGACCACCCATGAAATATGAGCT
KRAS_2_F	ACACTGACGACATGGTTCTACAGGTGGAGTATTTGATAGTGTATTAACC
KRAS_2_R	TACGGTAGCAGAGACTTGGTCTTAGCTGTATCGTCAAGGCAC
MIMIC_2_F	ACACTGACGACATGGTTCTACACCTGCTGTTCCTACAAAGCTG
MIMIC_2_R	TACGGTAGCAGAGACTTGGTCTAGGCTTCTCCCGTCTAAGGA

Note. Mimic amplicons contained the sequence context of KRAS codons 12 and 13, including 5 preceding and 3 subsequent nucleotides (total synchronous sequence: GAGCT GGTGGC GTA) at the same position within the amplicon. Sequence includes Fluidigm CS adapters.

F, forward primer; Number, amplicon number; R, reverse primer.

**Supplementary Table 5:** Polymerase Chain Reaction Components

Gene	Primers	dNTPs	5X Phusion HF Reaction Buffer (New England BioLabs),	Phusion High-Fidelity DNA Polymerase	Water
KDM6A	1 μM	0.5 mM	5 μL	1 U	To 25 μL
KRAS	1.2 μM pair 1 0.8 μM pair 2	0.5 mM	5 μL	1 U	To 25 μL

Note. For KDM6A, pre-amplification was performed in multiplex groups (group 1: primers 1, 4, 7…group 2: 2, 5, 8… group 3: 3, 6, 9…). For KRAS, each sample was amplified in 2 independent duplex polymerase chain reactions containing a KRAS and corresponding mimic primer pair.

**Supplementary Table 6:** Polymerase Chain Reaction Cycling Conditions Used for Amplification of KDM6A and KRAS

Step	Temperature	Time	Cycles
Initial denaturation	95 °C	2 min	1
Denaturation	95 °C	10 s	35
Annealing	60 °C	10 s	
Elongation	72 °C	15 s	
Final Elongation	72 °C	5 min	1

Note. Polymerase chain reaction was followed by ExoSAP-IT enzyme treatment (2 μL of enzyme for 5 μL of sample, ThermoFisher) at 37 °C for 15 minutes and 15-minute inactivation at 80 °C and 1:10 dilution in DNA Suspension Buffer (Teknova). For KDM6A, samples were further amplified using the Fluidigm Access Array according to the supplier’s protocol.

**Supplementary Table 7:** Summary of Mutations Identified in KDM6A^−^ Patches

Gender	Sample	Genomic coordinate	Exon	Ref	Alt	Alt freq	Maximum alt freq	Change
Male	1A	X:45078391	intron 18	A	G	85.8	100	Intronic
Male	1B	X:45078391	intron 18	A	G	72.9	100	Intronic
Male	2A	X:45078391	intron 18	A	G	82.9	100	Intronic
Male	2B	X:45078391	intron 18	A	G	85.3	100	Intronic
Male	3A	X:45085892	24	C	A	51	100	S1154>STOP
Male	3B	X:45085892	24	C	A	0	100	N/A
female	4A	X: 45089773	25	G	A	15.8	50	W1193>STOP
female	4B	X: 45089773	25	G	A	10.4	50	W1193>STOP

Note. Frequencies derived from frequency of mutant reads in samples. Of note, the mutation found in intron 18 was present in 2 independent patches. Furthermore, the mutations S1154∗ and W1193∗ have previously been found in cancer samples (COSMIC database).

Alt, alternative nucleotide -> this is the mutation; Change, predicted consequence of the mutation; freq., frequency; Ref, nucleotide in reference genome.

**Supplementary Table 8:** Sequence Context Used to Extract Mutant Reads From Sequencing Data

Context	Nucleotide change	Amino acid change
GAGCTGGTGGCGTA	WT	WT
GAGCTGATGGCGTA	G>A	G12D
GAGCTGCTGGCGTA	G>C	G12A
GAGCTGTTGGCGTA	G>T	G12V
GAGCTAGTGGCGTA	G>A	G12S
GAGCTCGTGGCGTA	G>C	G12R
GAGCTTGTGGCGTA	G>T	G12C
GAGCTGGTGACGTA	G>A	G13D
GAGCTGGTGCCGTA	G>C	G13A
GAGCTGGTGTCGTA	G>T	G13V
GAGCTGGTAGCGTA	G>A	G13S
GAGCTGGTCGCGTA	G>C	G13R
GAGCTGGTTGCGTA	G>T	G13C
GAGCTGGAGGCGTA	T>A	WT
GAGCTGGCGGCGTA	T>C	WT
GAGCTGGGGGCGTA	T>G	WT
GAGCTGGTGGAGTA	G>T	WT
GAGCTGGTGGGGTA	G>C	WT
GAGCTGGTGGTGTA	T>C	WT

Note. For each variant, the mutant allele frequency was calculated by dividing by the total read number.

## Mathematical Background

### Calculating the Crypt Fusion Rate

For individual crypts, fusion/fission events occur at a rate *ρ* and have a duration *Δτ* . If we take a snapshot of a piece of tissue at a time *t*_*0*_ we see all fusion/fission events that occurred in the window *[t*_*0*_ *− Δτ, t*_*0*_*]*. Calculating the average number of events per crypt, *X*, in a time *Δτ* over many snapshots is the same as calculating the probability of an event for a single crypt in the window *Δτ* (as we can only have a single event in any time window equal to the event duration). The number of events for a single crypt follows a Poisson distribution,

(1) $$X_{1}∼\text{Poi}\left(\rho \Delta \tau \right).$$

We want to observe events on the edge of mutant patches such that we can differentiate fission events from fusion events. For a patch with edge length *N* (that is, *N* crypts define the patch perimeter, each with at least one wild-type crypt as a neighbor), the number of crypts undergoing fusion or fission is distributed as

(2) $$X_{\text{N}}∼\text{Poi}\left(N\rho \Delta \tau \right).$$

For a given patch with edge length *N*, then, the probability of zero events in a window *Δτ* is

(3) $$p\left\{X_{N}=0\right\}={\text{e}}^{-N\rho \Delta \tau }∼1-N\rho \Delta \tau +ο\left(N^{2}\rho^{2}\Delta \tau^{2}\right),$$

where *Nρ* is considered small compared with *Δτ* such that we may define a parameter *ε = NρΔτ* where *ε<<1*. Correspondingly, the probability of seeing at least 1 event in a window *Δτ* is

(4) $$p\left\{X_{N}\geq 1\right\}=1-p_{0}∼N\rho \Delta \tau +ο\left(\varepsilon^{2}\right).$$

This equation can be applied to either fusion events or fission events separately by changing the event rate *ρ* to *ρ*_*fu*_ or *ρ*_*fi*_, the event rates of fusion and fission, respectively, assuming that the event duration *Δτ* is approximately equal for fission and fusion.

#### Calculating the fusion rate

Let the number of partially mutant (partial) and fully mutant (monoclonal) FUFI events observed on the edge of mutant patches be *n*_*p*_ (termed M/W in main text) and *n*_*m*_ (termed M/M in main text), respectively. These numbers are combined over many different mutant patches and tissue samples, with a total patch edge length of *N*. To calculate the fusion rate *ρ*_*fu*_ given the fission rate *ρ*_*fi*_, we use the following observations and assumptions:

1. All fission events are monoclonal.
2. Not all monoclonal events are fissions.
3. All partial events are fusions.
4. Not all fusions are partials.

The first and third assumptions stem from the belief that the timescale over which monoclonal conversion occurs in a crypt is short compared to the time between fusion/fission events. The second and fourth observations are alternative statements of the fact that fusion at the patch edge can happen inwards: a mutant crypt on the patch edge can fuse with a mutant crypt within the patch, hence creating a monoclonal event. We define the parameter *χ* to be the proportion of fusion events that are M/M. Then the ratio *n*_*p*_/*n*_*m*_ may be written as

(5) $$\frac{n_{p}}{n_{m}}=\frac{2n_{\text{fu}}\left(1-x\right)}{n_{\text{fi}}+2n_{\text{fu}}x},$$

where n_fu_ and n_fi_ are the number of fusion and fission events, respectively. The factor of 2 accompanying each instance of *n*_*fu*_ in (5) comes from the hypothesis that fusion can be initiated by either of the participating crypts, so the number of events associated with the fusion rate of any individual crypt should be half the number observed. We do not know *n*_*fu*_ and *n*_*fi*_ a priori, however.

We may recast the probability *p{X*_*N*_ *≥ 1}* from (4) as the number of observed events over sample size, *n*_*events*_*/N*, for fusion and fission, we get

(6) $$\frac{n_{\text{fu}}}{N}∼N_{\rho \text{fu}}\Delta \tau ,$$

and

(7) $$\frac{n_{\text{fi}}}{N}∼N_{\rho \text{fi}}\Delta \tau ,$$

where we have assumed the duration of a fusion event is approximately equal to that of a fission event. Thus, we find the approximate equivalence

(8) $$\frac{n_{\text{fi}}}{n_{\text{fu}}}∼\frac{\rho_{\text{fi}}}{\rho_{\text{fu}}}$$

between the ratios of numbers and rates of events. Using this, we may rewrite (5) as

(9) $$\frac{n_{p}}{n_{m}}=\frac{2\left(1-x\right)}{\frac{\rho_{\text{fi}}}{\rho_{\text{fu}}}+2x},$$

and subsequently find an expression for the fusion rate

(10) $$\rho_{\text{fu}}=\frac{\rho_{\mathrm{fi}}}{2\frac{n_{m}}{n_{p}}\left(1-x\right)-2x}.$$

The simplest model for the parameter *χ* is to assume (isotropic) fusion, such that the proportion of fusions that are monoclonal is simply

(11) $$x=\frac{N_{t}-N_{\omega }}{N_{t}},$$

where *N*_*t*_ and *N*_*w*_ are the total number of neighbors and number of wild-type neighbors of a given fusion event, respectively. To get a bulk measure of *χ* for each clonal mark, *N*_*t*_ *and N*_*w*_ were averaged over all observed M/M events.

### Diffusion Model of Tissue Reorganization

In the following we lay out the theoretical framework and statistical methods used for understanding tissue rearrangement due to clonal expansion in the gut as a diffusion process.

#### Parameterization and derivation

We approach the idea of crypt packing by defining a quantity *γ* that represents the local stromal fraction of the tissue (we also refer to this as the “white space” fraction); then, the crypt area per unit area of mucosa is given by *1-γ*. If you look at a small region of tissue in cross-section, *γ* is the fraction of that region that is taken up by stroma rather than epithelial cells (ie, that fraction that is not part of a crypt). It is useful to think of the crypts as a density that is moving in the “free space” of the stroma. Then, we can define another quantity ψ that represents this density in such a way that *ψ ≡ 1/γ*. This density *ψ* tells us the number of units of area we would need to observe to see 1 unit of area of white space. This is a convenient definition, as it allows the density to be expressed as a function unbounded in the positive real numbers, whereas the local crypt fraction *1-γ* is bounded in [0,1].

In intestinal tissue, we assume there is a patient-specific homeostatic degree of crypt packing that can be represented by “ambient” values *γ*_*a*_, *ψ*_*a*_ for the stromal fraction and crypt density, respectively. Near to a region of clonal expansion (a fission-driven mass source), the tissue is perturbed and the crypt density is altered such that

(12) $$\psi \left(\text{r,t}\right)=\psi_{\text{a}}+\tilde{\psi }\left(\text{r,t}\right),$$

where the spatiotemporal variation in the crypt density is contained in the perturbation term *ψ̃(r, t)*. By centering our polar coordinate system *r = (r, θ)* at the initiation point of the clonal expansion we can state our far-field condition: *ψ̃ → 0* as *r → ∞*, meaning that we expect the tissue to remain in its ambient structure far from any perturbation. We choose to model the density perturbation *ψ̃* as undergoing diffusive dynamics governed by the 2D diffusion equation

(13) $$\frac{\partial \tilde{\psi }}{\partial t}=\nabla \cdot \left(D\nabla \tilde{\psi }\right),$$

where the coefficient of diffusion *D* quantifies the speed with which the tissue can react to new mass being created by fission by rearranging to accommodate it. We assume *D* is isotropic and homogeneous such that (13) simplifies to

(14) $$\frac{\partial \tilde{\psi }}{\partial t}=\nabla \cdot \left(D\nabla \tilde{\psi }\right).$$

We want to use (14) to understand the dynamical process underlying observed patches of clonally expanded mutant tissue; we note that although we know the initial size (a single crypt) and the current size (*n* mutant crypts) of the of the clonal expansion, we do not know the total age of the mutant patch or when the individual fission events driving the expansion occurred. We first show how to solve the system in the case of an instantaneous injection of mass at time *t=0* before showing how to use this solution to build a more realistic model of dynamic fission events.

We approach (14) by taking a spatial Fourier transform such that the transformed density

(17) $$\hat{\psi }\left(k_{x},k_{y},t\right)=\int_{-\infty }^{\infty }\int_{-\infty }^{\infty }\tilde{\psi }\left(x,y,t\right){\text{e}}^{-ik_{x}x_{\text{e}}-ik_{y}y\;}\text{d}x\text{d}y,$$

is governed by

(18) $$\frac{\partial \hat{\psi }}{\partial t}=-D\left(k_{x}^{2}+k_{y}^{2}\right)\hat{\psi },$$

where *k = (k*_*x*_*, k*_*y*_*)* is the wave vector in a Cartesian coordinate system *(x, y)* with its origin at the initiation point of clonal expansion. Equation (18) can be solved by integrating with respect to time and applying the initial condition *ψ∧(0) = F [M δ(r)]*, where M is the extra crypt area introduced by the injection of mass. We find

(19) $$\hat{\psi }\left(\text{k},t\right)=M{\text{e}}^{-D|\text{K}|{|}^{2}t}.$$

We proceed by inverting the Fourier transform to find the crypt density in terms of the spatial coordinates *x* and *y*. First, note that the inversion can be separated as follows:

(20) $$\psi \left(x,y,t\right)=M{\left(\frac{1}{2\pi }\right)}^{2}\left(\int_{-\infty }^{\infty }e^{-Dk_{x}^{2}t+ik_{x}x\;}dk_{x}\right)\left(\int_{-\infty }^{\infty }e^{-Dk_{y}^{2}t+ik_{y}y\;}dk_{y}\right).$$

Completing the square in the exponent of the *x* integrand we can recast the integral as

(21) $$\int_{-\infty }^{\infty }e^{-Dk_{k}^{2}t+ik_{x}x\;}dk_{x}=e^{-\;\frac{x^{2}}{4Dt}}\int_{u\left(-\infty \right)}^{u\left(\infty \right)}e^{-Dtu^{2}}du,$$

where *u = k*_*x*_ *+ ix/2Dt* and the integral on the right hand side of (21) can be evaluated:

(22) $$\int_{u\left(-\infty \right)}^{u\left(\infty \right)}e^{-Dtu^{2}}du=\sqrt{\frac{\pi }{Dt}.}$$

By symmetry, the full solution is found to be

(23) $$\psi \left(r,t\right)=\frac{M}{4\pi Dt}e^{-\frac{r^{2}}{4Dt}},$$

where r^2^ = x^2^ + y^2^ is the radial distance from the center of the clone. The stromal fraction near to a mutant patch may be expressed using (23) as

(24) $$\gamma \left(r,t\right)=\frac{1}{\psi_{a}+\psi \left(r,t\right)}=\frac{\gamma_{a}}{1+\frac{M\gamma_{a}}{4\pi Dt}E_{d}\left(r\right)},$$

where for notational ease we have defined the exponential function

(25) $$E_{d}\left(x\right)=e^{-\;\frac{x^{2}}{4Dt}.}$$

#### Diffusion with a stochastically firing point source

To investigate the temporal aspect of clonal expansion, the diffusion model was extended to accommodate growth over time due to stochastic crypt fission at the patch center. One can intuitively think of this as overlaying identical diffusion processes in space with different initiation times. Mathematically, this can be achieved by breaking the solution space into chunks separated by each fission event time. If the Fourier-space density *ψ∧*_*1*_*(t)* is the solution of (18) in a time interval *[t*_*0*_ *, t*_*1*_*)*, achieving a value *ψ∧*_*1*_*(t*_*1*_*)* at the end of this period, then the solution *ψ∧*_*2*_*(t)* for the next period *[t*_*1*_*, t*_*2*_*)*, where a fission event occurs at *t*_*1*_, is found by solving (18) with the initial condition *ψ∧*_*2*_*(t*_*1*_*) = ψ∧*_*1*_*(t*_*1*_*) + F [a*_*m*_ *δ(r)]* where *a*_*m*_ is the area of the new mutant crypt. Performing this process iteratively produces, with initial area perturbation *A*_*0*_ *= a*_*m*_ *− a*_*w*_ generated by the mutational hit, the compound diffusion solution

(26) $$\psi \left(k,t\right)=A_{0}e^{-D{\left|K\right|}^{2}t}+\sum_{i=1}^{n_{f}}a_{m}e^{-D{\left|K\right|}^{2}\tau i},$$

where τ_i_ = t − Σ_j_^i^ t_j_ for *i ∈ [1, n*_*f*_*]* with *t*_*k*_ the event times of *n*_*f*_ crypt fissions. Inverting the Fourier transform gives the full spatial solution for the density perturbation:

(27) $$\psi \left(r,t\right)=\frac{A_{0}}{4\pi Dt}e^{-\;\frac{r^{2}}{4Dt}}+\sum_{i=1}^{n_{f}}\frac{a_{m}}{4\pi D\tau_{i}}e^{\frac{r^{2}}{4Dr_{i}}},$$

The formulation (27) supposes that we do not know how large a mutant patch will grow in a given amount of time t, but that we can generate fission times *t*_*k*_ and take those events with *t*_*k*_ *< t* to define our solution up to the desired time *t*. We can so generate fission times given a fission rate *ρ* by assuming exponential waiting times (between events generated by a Poisson process) and using

(28) $$t_{n}=-\frac{\ln\left(1-u_{01}\right)}{\rho n},$$

where *n* is the current patch size, *t*_*n*_ is the time of the nth fission event, and *u*_*01*_ is a random draw from the unit uniform distribution *U(0, 1)*. This method can also be used to generate mutation times using the mutation rate *ɑ* (9.3 x 10^−7^ for KDM6A; 1.7 x 10^−6^ for STAG2), the monoclonal accumulation rate *ΔC* (6.0 x 10^−6^ for KDM6A; 2.1 x 10^−5^ for STAG2), the number of stem cells per crypt undergoing neutral drift *N*_*stem*_ *(7)* and the total number of crypts in the gut *N*_*crypts*_ (∼10 x 10^6^). So given a mutation that confers an expansion bias in terms of an increased fission rate over wild type epithelium, we can simulate the distribution of theoretical clonal expansion dynamics by generating a mutation time and a sequence of fission event times using (28) and calculate the evolution of the packing density over time using (27). The full theoretical white space fraction is given by inserting (28) into

(29) $$\gamma \left(r,t\right)=\frac{1}{1/\gamma_{a}+\psi \left(r,t\right)}.$$

#### Comparing theory to experiment

Although (29) fully describes the theoretical diffusion process we posit as an explanation for the alleviation of crypt packing in the gut in lieu of mass crypt fusion, we must do more work to form a quantity suitable for comparison with experimental measurements. The data we have are measurements of the total area of patches of *n* crypts (including their “share” of the stromal space bordering the patch) and the area of the individual crypts making up each patch. Thus we can calculate the total white space *Γ* in a patch by subtracting the summed crypt areas from the total patch area. We can get a comparable theoretical quantity *Γ*_th_ by integrating the theoretical white space fraction (29) over the area *A* occupied by a patch *P* of *n* crypts:

(30) $$\Gamma_{th}=\iint_{PA}\;\gamma \left(r,t\right)dA.$$

The integral (30) is nontrivial for an arbitrary patch geometry. To simplify the problem, we transform the geometry of the patch into a subsector of a circle centered at the coordinate origin while conserving the patch area.

To do this, 3 quantities must be found: the angle *θ*_*s*_ subtended by the subsector, its inner radius *R*_*in*_, and its outer radius *R*_*out*_ . To find *θ*_*s*_ the distance d between the centroid of the patch and the centroid of the mutant source patch (the coordinate origin) is first calculated. Then,

(31) $$\theta_{8}=\left\{ \begin{array}{l} 2a\;\tan\frac{R_{w}}{d},\;\text{if }d>0,\\ 2\pi ,\;\text{if}\;d=0, \end{array}\right.$$

where *R*_*w*_ is the radius of the patch being transformed (and where “radius” means the radius that would produce the area of the patch if the patch were a circle). Now, the area of a subsector is given by

(32) $${A\;}_{\sec}=\frac{\theta_{8}}{2}\left(R_{out}^{2}-R_{in}^{2}\right),$$

and area conservation enforces the equality *A*_*sec*_ *= πR*_*w*_^*2*^. If *d > 0* we may use the approximation *R*_*in*_ *= d − cR*_*w*_ and *R*_*out*_ *= d + cR*_*w*_ for some small *c > 0* and substitute into (32) to find

(33) $$c=\frac{\pi R_{W}}{2d\theta_{s}}.$$

Using (31) to fix the value for R in as

(34) $$R_{in}=\left\{ \begin{array}{l} d-\frac{\pi R_{w}^{2}}{2d\theta_{s}},\;\text{if}\;d>0,\\ 0,\;\text{if}\;d=0, \end{array}\right.$$

we may then invoke area conservation once more to find

(35) $$R_{out}=\sqrt{R_{in}^{2}+\frac{2\pi R_{W}^{2}}{\theta_{s}}}.$$

We can now approximate the integral (30) with

(36) $$\Gamma_{th}\approx \int_{0}^{\theta }\int_{R_{in}}^{R_{out}}\gamma \left(r,t\right)rdrd\theta ,$$

which we evaluate numerically. The theoretical value for the total patch white space defined in (36) can then be used to fit the diffusion model given a data set of patch measurements.

#### Statistical model for inferring the diffusion coefficient

To infer the parameters defining the tissue-intrinsic diffusion process, we define the likelihood as

(37) $$\Gamma^{\left(pq\right)}∼N\left(\Gamma_{th}^{\left(pq\right)}\left(D,r_{a}^{\left(p\right)},t^{\left(p\right)},m^{\left(p\right)},L^{\left(pq\right)}\right),\sigma \Gamma \right),$$

where *Γ*^*(pq)*^ and *Γ*_*th*_ are respectively the observed and theoretical total white space in patch *q* of the neighborhood of the *p*th mutant patch (as defined in [36]). The parameters defining *Γ*^*(pq)*^_*th*_ are the single tissue-intrinsic diffusion coefficient *D*, the ambient stromal fraction *γ*_*a*_, sequence of event times (mutation and subsequent fissions) *t*^*(p)*^, and input areas *M*^*(p)*^ (mutant and wild type crypt areas *a*_*m*_*, a*_*w*_) of the *p*th mutant patch, and the location parameters *L*^*(pq)*^ which define the transformed inner and outer radii and angle subtended by the patch *q* of the neighborhood of the *p*th mutant patch. We simultaneously infer the coefficient of diffusion *D* and the local ambient white spaces *γ*_*a*_.

We use a hierarchical model to constrain *γ*_*a*_ such that each value is drawn from a population distribution

(38) $$\gamma_{a}^{\left(p\right)}∼\mathrm{Beta}\left(\alpha_{pop},\beta_{pop}\right),$$

with hyperpriors

(39) $$\alpha_{pop}∼\mathrm{Gamma}\left(10,1\right),\;\beta_{pop}∼\mathrm{Gamma}\left(10,1\right),$$

chosen to be uninformative around an unbiased mean value *γ*_*a*_^pop^ = 1/2. Standard normal distributions bounded to the positive real line are used as prior distributions for the diffusion coefficient and the standard deviation *σ*_*Γ*_ about the theoretical total white space,

(40) $$D∼N^{\left(+\right)}\left(0,1\right),\;\sigma \Gamma ∼N^{\left(+\right)}\left(0,1\right).$$

Inference for the full model defined by equations (37) to (40) was performed by MCMC sampling using the Stan probabilistic programming language. The sequence of event times *t*^*(p)*^ an input to the model, generated using (28). For each clone 1000 potential sequences (trajectories) were generated and the inference was run 250 times with a random sample of the potential trajectories used each time. This allowed us to be sure that the resulting model parameters accounted for the large variation in the temporal development of the patches due to the stochastic nature of the mutational hit and the fissions themselves. From the resulting distributions, “most likely” trajectories were selected by fixing the diffusion coefficient D and ambient stromal fraction of each neighborhood *γ*_*a*_ to their median values and evaluating the likelihood of the data under the model for each of the 1000 potential trajectories per clone. For each clone, the 25 most likely trajectories were selected and averaged to give an approximation of the likely age of each patch.

To test whether we are justified in imposing the form of a diffusion process on the data we performed the inference above for a null model wherein each neighborhood around a mutant clone would be explained by a constant stromal fraction. This involved inferring *γ*_*a*_ for each patch and setting *γ(r) = γ*_*a*_, and then calculating *Γ*^*(pq)*^_*null*_ and using it in (37) to evaluate the likelihood of the data under the null model. To compare the diffusion model to the null model, the full set of 1000 potential trajectories for each clone were used as input to calculate a distribution of model likelihoods given the inferred population median diffusion coefficient and the per-clone median ambient stromal fraction. The results show that there is more evidence to support the hypothesis of identifying the mutant patch as the source of clonal expansion causing a diffusion-like radial dependence in the local stromal fraction.

#### Polyp initiation

Here we quantify when the biological process might break down due to physical constraints on crypt density. In the theoretical scheme defined previously, as crypt density *ψ → ∞* the stromal fraction *γ → 0*. Below some local value of *γ*, there will not be enough space for a crypt to fission. We posit that this may be a mechanism for outward growth, or polyp formation. A useful threshold to set on the available white space can be borrowed from the mathematics of optimal packing; identical circles may be optimally hexagonally packed to fill a fraction *π/√12* of the space. Thus, we set the lower bound on the stromal fraction *γ*_*b*_ *= 1 − π/√12*. A mutant patch that creates mass through fission at such a rate that the tissue-intrinsic diffusion cannot act to fully accommodate new crypts will have a decrease over time of the available stromal fraction as the patch grows exponentially. We claim that if the available white space dips below *γ*_*b*_ then there the patch has a finite chance of initiating outgrowth.

We can quantify this more concretely by simulating an ensemble of mutant patches with a given fission rate. At each time point, we can find the radial extent *r*_*i*_ of each instance *i* of the mutant patch by constraining the integral over the nonstromal fraction to equal the total area of the *n*_*i*_ mutant crypts in the patch,

(41) $$\int_{0}^{2\pi }\int_{0}^{r_{i}}\left(1-\gamma \left(r\right)\right)rdrd\theta =n_{i}a_{m},$$

where the mutant crypts have area *a*_*m*_. To approximate the effective local stromal fraction throughout the patch, the cumulative average of *γ(r)*, *γ̄(r)* is calculated starting from the patch center *r = 0*. For each patch area *πr*_*i*_^*2*^ we may then calculate the fraction *p*_*f*_ of this area for which *γ̄ < γ*_*b*_ . This gives us an idea of the portion of the patch that is at risk of initiating a polyp with the next fission event. Averaging *p*_*f*_ over tsssshe whole ensemble, we find an estimate for the probability of a clonal expansion developing into a polyp for a given time *t* after the initial mutation.
